# Effects of physiotherapy on degenerative cerebellar ataxia: a systematic review and meta-analysis

**DOI:** 10.3389/fneur.2024.1491142

**Published:** 2025-01-10

**Authors:** Akiyoshi Matsugi, Kyota Bando, Yuki Kondo, Yutaka Kikuchi, Kazuhiro Miyata, Yuichi Hiramatsu, Yuya Yamanaka, Hiroaki Tanaka, Yuta Okuda, Koshiro Haruyama, Yuichiro Yamasaki

**Affiliations:** ^1^Faculty of Rehabilitation, Shijonawate Gakuen University, Osaka, Japan; ^2^National Center Hospital, National Center of Neurology and Psychiatry, Tokyo, Japan; ^3^Department of Rehabilitation for Intractable Neurological Disorders, Institute of Brain and Blood Vessels Mihara Memorial Hospital, Gunma, Japan; ^4^Department of Physical Therapy, Ibaraki Prefectural University of Health Science, Ibaraki, Japan; ^5^Neurorehabilitaion Research Institute, Morinomiya Hospital, Osaka, Japan; ^6^Department of Rehabilitation, Osaka University Hospital, Osaka, Japan; ^7^Department of Physical Medicine and Rehabilitation, Kansai Medical University Hospital, Osaka, Japan; ^8^Department of Physical Therapy, Faculty of Health Science, Juntendo University, Tokyo, Japan; ^9^Department of Rehabilitation, Maruki Memorial Medical and Social Welfare Center, Saitama, Japan

**Keywords:** cerebellum, ataxia, degenerative cerebellar ataxia, physical therapy, physical rehabilitation, systematic review, meta-analysis

## Abstract

**Background:**

Evidence of the effectiveness of physiotherapy, including muscle strength training, coordination training, aerobic exercise, cycling regimen, balance training, gait training, and activity of daily living training, in patients with degenerative cerebellar ataxia (DCA) was insufficient for clinical decision making. We aimed to explore clinical outcomes and examine the parameters associated with physical impairment and activity in people with DCA based on preregistration (PROSPERO: CRD42024493883).

**Methods:**

The PubMed, Cochrane Library, CHINAL, and PEDro databases were searched for relevant randomized controlled trials (RCTs). Data extraction, quality assessment, and heterogeneity analyses were conducted. The Grading of Recommendations Assessment, Development, and Evaluation framework (GRADE) was used to assess the quality of evidence, and a meta-analysis was performed.

**Results:**

Eighteen RCTs, which included 398 participants, showed a serious risk of bias (RoB) and low certainty of evidence for this primary outcome. For meta-analysis, 315 patients assessed based on the Scale for Assessment and Rating of Ataxia (SARA) were included. Overall, physiotherapy significantly reduced SARA scores (MD = −1.41, [95% CI: −2.16, −0.66]); the subgroup analysis showed that the following interventions exerted significant effects: multi-aspect training program (5 studies, MD = −1.59, [95% CI: −5.15, −0.03]), balance training (3 studies, MD = −1.58, [95% CI: −2.55, −0.62]), and aerobic training (3 studies, MD = −1.65, [95% CI: −2.53, −0.77]). By contrast, vibration (2 studies, MD = −0.56, [95% CI: −2.05, 0.93]) and dual-task training (1 study, MD = 0.24, [95% CI: −6.4, 6.88]) exhibited no significant effects.

**Conclusion:**

Physical therapy, especially multi-aspect physical therapy such as muscle strengthening, coordination training, gait training, and ADL training, may reduce DCA symptoms. Further, balance and aerobic training can be added to the program. However, the estimated effect size may change in future studies because of the serious RoB, very low certainty of evidence, and high heterogeneity with SARA as the primary outcome. High-quality RCTs are required to establish evidence for the effectiveness of physical therapy in patients with DCA.

**Systematic review registration:**

https://www.crd.york.ac.uk/prospero/display_record.php?RecordID=493883, identifier: CRD42024493883.

## 1 Introduction

Degenerative cerebellar ataxia (DCA) includes various neurodegenerative disorders characterized by progressive cerebellar dysfunction and Purkinje cell loss, leading to cerebellar atrophy (1). Degeneration of the cerebellum, brainstem, or spinal cord can induce diverse clinical symptoms. Limited treatment options improve daily activities and quality of life (QOL), highlighting the need for novel, safe, and effective non-pharmacological interventions (2, 3). Physical therapy (PT) and neurorehabilitation have shown potential as interventions for cerebellar ataxia (4, 5), but the precise effect estimates and the certainty of their effectiveness have not been thoroughly evaluated.

The effects of PT on DCA have been examined in randomized controlled trials (RCTs) and systematic reviews (5). The most recent systematic review included eight articles for meta-analysis, of which six examined the effects of PT on the Scale for Assessment and Rating of Ataxia (SARA) as the primary clinical outcome for DCA (6, 7). However, one was not an RCT and two did not focus on spinocerebellar disease (SCD). Previous studies examining the effects of therapeutic exercise on cerebellar ataxia identified several limitations, including variability in the control groups used. First, the quality of included studies varies widely, which may affect result reliability. Specifically, the quality of evidence regarding functional independence is low, making the conclusions difficult to generalize. Second, studies are focused on non-hereditary degenerative and acquired cerebellar ataxia; data on hereditary cerebellar ataxia remain sparse. Additionally, the sample sizes in these studies are often small, and the treatment durations are short, limiting the ability to evaluate long-term effects. Last, the reported results show inconsistencies and potential for bias, particularly in non-randomized studies.

Another critical limitation of previous studies is the variability in the control groups used. While some studies utilized passive controls (e.g., no intervention), others employed active controls (e.g., alternative physiotherapy methods or standard care). This distinction is particularly important as the use of active control groups is increasingly common due to ethical considerations in rehabilitation trials, where withholding treatment from control participants may be deemed inappropriate (8). However, this trend complicates the interpretation of findings and the synthesis of results in meta-analyses, as the comparator conditions can substantially influence the observed treatment effects. A clearer understanding of the relative effectiveness of interventions under different control conditions is essential for clinical decision-making.

These limitations highlight the need for high-quality, large-scale studies to clarify the benefits of therapeutic exercise in this population. Several RCTs have been conducted since then to address the issues raised in this systematic review. However, a new systematic review updating the effect estimates for PT and showing improved certainty is lacking.

Thus, this systematic review and meta-analysis aimed to investigate the effects of a multi-aspect PT program, including strength training, coordination training, aerobic exercise, balance training, gait training, activity of daily living (ADL) training, and vibration stimulation, on SARA as the primary outcome of ataxia severity. In addition, we included the following secondary outcomes unaddressed in previous systematic reviews: International Cooperative Ataxia Rating Scale (ICARS) (6), Berg Balance Scale (BBS) (9), Balance Evaluation Systems Test (BESTest) (10, 11), functional independence measure (FIM) (12), QOL-related indicators (13), and gait ability. Furthermore, we analyzed the results separately for passive and active control groups to better understand the impact of different comparators on treatment outcomes. The findings of this study are expected to contribute to future research questions and decision making for clinical interventions.

## 2 Methods

### 2.1 Overall

This systematic review was conducted in accordance with the guidelines in the Preferred Reporting Items for Systematic Reviews and Meta-Analysis (PRISMA) statement (see Supplementary material “PRISMA checklist”) (14). The protocol was registered in the International Prospective Register of Systematic Reviews (PROSPERO) database (ID: 2023 CRD42023379192).

### 2.2 Eligibility criteria

The inclusion criteria for selecting studies in this review included the following: (1) randomized controlled trials (RCTs), (2) participants with DCA, (3) the use of PT as an intervention, and (4) articles written in English. The exclusion criteria were as follows: (1) studies that were not RCTs and (2) conference papers, protocol papers, or registration reports.

DCA comprises a diverse range of disorders, which include autosomal dominant spinocerebellar ataxia (15), spinocerebellar ataxia (SCA) (16–18), Friedreich's ataxia (FA) (19), multiple system atrophy with cerebellar involvement (20), and sporadic adult-onset ataxia of indeterminate cause (21). Given this heterogeneity, our systematic review intentionally broadened its scope beyond any single phenotype, such as SCA, to ensure a comprehensive analysis.

### 2.3 Information sources and search strategy

We searched the PubMed, Cochrane Central Register of Controlled Trials, CINAHL, and PEDro databases for studies published in English and involving human participants. We developed a search query for these databases (Appendix 1). The search was performed on March 8, 2024 and included all articles published up to that date.

### 2.4 Article selection

The search was conducted by independent reviewers (Akiyoshi Matsugi and Hiroaki Tanaka) using the specified databases, and the initial list of articles was verified by other reviewers. In addition, manual searches were performed with relevant keywords such as “cerebellum,” “spinocerebellar degeneration,” “ataxia,” “physiotherapy,” and “rehabilitation.” The studies identified in the databases were managed using Rayyan (Cambridge, MA) and ENDNOTE 20 (Clarivate, Philadelphia, PA).

### 2.5 Data collection

For each study, the two independent reviewers, selected at random from a pool of 11 individuals (Akiyoshi Matsugi, Kyota Bando, Yuki Kondo, Yutaka Kikuchi, Kazuhiro Miyata, Yuichi Hiramatsu, Yuya Yamanaka, Hiroaki Tanaka, Yuta Okuda, Koshiro Haruyama, and Yuichiro Yamasaki), were tasked with screening the titles and abstracts to assess eligibility for inclusion. Full-text assessments were undertaken when deemed necessary. Initially, the reviewers were blinded to each other's identities to mitigate potential biases, and any discrepancies in judgment were adjudicated by a third reviewer. The identities of the reviewers were disclosed during the final deliberation to ensure transparency. Extraction of data, encompassing study design, methodological approach, participant demographics, baseline characteristics, sample sizes, and outcome measures, was independently conducted by the two reviewers. Any inconsistencies in data extraction were resolved through consultation with a third reviewer. In case of missing data, corresponding authors were contacted; if responses were not received or data were not provided, analyses were confined to the available data. Extracted data were systematically organized using a Microsoft Excel spreadsheet.

### 2.6 Data items

We assessed ataxic symptoms using SARA as the primary outcome (6). The use of this scale is recommended for assessing cerebellar ataxia as a clinician-reported outcome measure (22). The secondary outcomes included ICARS (23), gait speed, dynamic gait index (DGI) (24), FIM (25), Inventory of Non-Ataxia Signs (INAS), Euro Quality of Life Visual Analog Scale (EQ-VAS), BBS, and other reported outcomes that the reviewers considered important. Other additional important outcomes selected by the reviewers included fall frequency, Activities of Balance Confidence questionnaire (ABC), functional ambulatory capacity (FAC) (26), 8-meter walk test (8MWT), timed up and go test (TUG), modified Clinical Test Sensory Interaction and Balance (mCTSIB), 9-hole peg test (9HPT), Barthel index (BI), MOS 36-Item Short-Form Health Survey (SF-36), Euro quality of life 5 dimension (EQ-5D), and Friedreich's Ataxia Rating Scale (FARS).

The weighted mean difference and the mean and standard deviations (SDs) were used for continuous data in the primary and secondary outcomes. The mean difference was used to summarize multiple measures of the same outcome items.

### 2.7 Study risk of bias assessment

The risk of bias (RoB) was evaluated using the Cochrane RoB tool (version 2.0) (27). Two out of five independent reviewers conducted a critical appraisal of the studies included in the analysis. The assessment focused on the following areas: (1) bias originating from randomization; (2) bias resulting from deviations from the intended interventions; (3) bias due to incomplete outcome data; (4) bias related to the assessment of outcomes; and (5) bias stemming from the selection of reported results. Each study was classified for each domain as having low, some concern, or high RoB. An algorithm-based approach, guided by responses to signaling questions, was employed to judge the RoB for each domain (27). Any disagreements among the reviewers were discussed If a consensus could not be reached, a third reviewer was consulted to resolve the issue.

### 2.8 Effect measures and synthesis methods

The primary outcome (SARA) and secondary outcomes (ICARS, BBS, INAS, gait speed, DGI, FIM, and EQ-VAS) were obtained as the mean of the pre-post difference (MD) and SD. The effect sizes were the MD and 95% confidence interval (CI) integrated using RevMan 5.4 for all outcomes.

If more than two randomized (or quasi-randomized) controlled trials reported the same outcomes, the weighted mean difference was calculated using RevMan 5.4 software. Random-effects models were used to obtain pooled estimates, and the results were described using forest plots in RevMan 5.4. If the MD and SD were obtained from the original report, we requested the authors for the data via email. Further, if we could not obtain the SD from the authors, missing SD of MD was calculated using the standard error (SE) or 95% CI. If the MD could not be obtained, we declined to integrate the data from that study into the MA.

To examine the effects of PT, we conducted a meta-analysis without separating subgroups. Subsequently, subgroup analysis was performed according to the type of intervention, which was divided into multiaspect PT, balance training, aerobic exercise, vibration, and dual-task physiotherapy.

### 2.9 Reporting bias assessment

Funnel plots were used to determine publication bias.

### 2.10 Certainty assessment

The overall quality of the evidence for all outcomes was appraised using the Grading of Recommendations Assessment, Development, and Evaluation (GRADE) (28) framework. This assessment encompassed several key factors: (1) study design, (2) RoB, (3) inconsistency of results, (4) indirectness of evidence, (5) imprecision of estimates, and (6) additional considerations (28). These elements were utilized to gauge the certainty of the effect estimates, classifying the quality of evidence into one of four categories: “very low,” “low,” “moderate,” and “high” (28).

## 3 Results

### 3.1 Study selection

**Figure 1:**
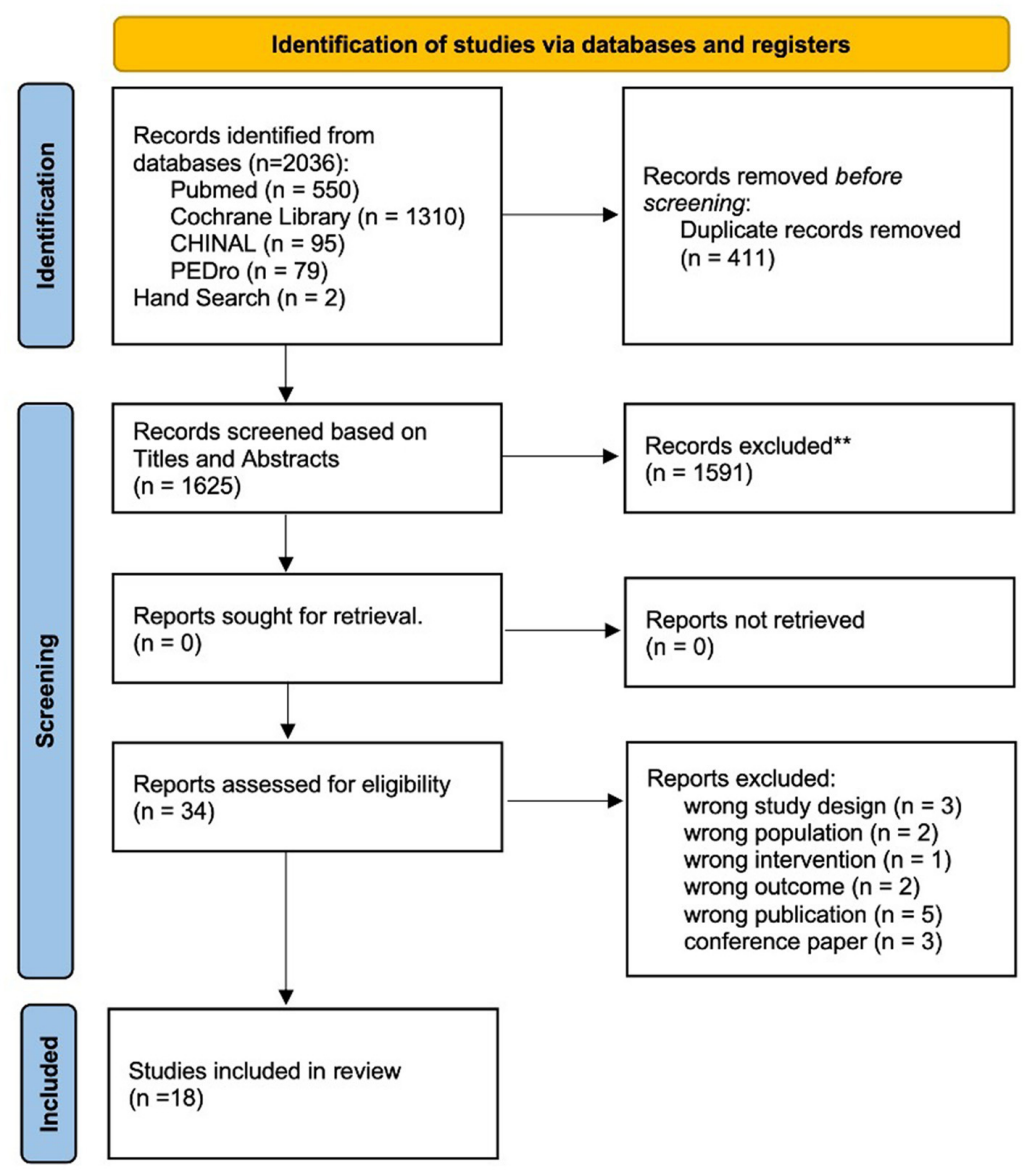
Flowchart depicting the article search and selection process according to the PRISMA guidelines. This diagram illustrates the steps taken to identify and screen articles, culminating in the selection of 18 studies that met the inclusion criteria for the systematic review. ^**^Indicates using Rayyan.

A flowchart of the selection process is shown in Figure 1. The review process was documented using the PRISMA checklist (Appendix 2).

A total of 2,036 articles were retrieved using a database search and additional records. After duplicate elimination, the titles and abstracts of 1,625 publications were selected. Among these, 34 articles underwent full-text screening for eligibility, and 16 articles were excluded based on the following criteria: (1) non-RCT study design (*n* = 3); (2) non-SCD population (*n* = 2); (3) physiotherapy was not the intervention used (*n* = 1); (4) outcome measure did not include symptoms associated with cerebellar ataxia (*n* = 2); (5) protocol paper (*n* = 5); and (6) conference paper (*n* = 3). Finally, 18 articles (4, 29–45) met the inclusion criteria and were included in the meta-analysis if outcome data were obtained from the publication or authors.

### 3.2 Study characteristics

The characteristics of the included studies are listed in Table 1. A total of 598 participants were involved in the 18 studies. The most common SCA subtypes were SCA6 (*n* = 48), SCA3 (*n* = 35), SCA2 (*n* = 19), SCA7 (*n* = 18), SCA1 (*n* = 16), and SCA31 (*n* = 6). Thirty-eight patients with FA were included. Many cases with unclear pathology were also included. All included studies reported no adverse side effects of physiotherapy. Funding information was insufficient in five studies (29, 36–38, 40). Active control intervention was applied to the control group in seven studies (29–31, 35, 38, 43, 44). Six studies examined the effects of comprehensive interventions that included multiple aspects of PT (4, 37, 39–42).

**Table 1:** Characteristics of included studies.

**No**.	**References**	**Study design**	**Participant**	**Age and baseline severity**	**Test of multi or single aspect of physiotherapy intervention**	**Intervention**	**Intensity, frequency, duration (for meta-analysis)**	**Control**	**Outcome**
1	Miyai et al. (4)	RCT	SCA6 (*n* = 20), SCA31 (*n* = 6), Idiopathic DCA (*n* = 16)	Age: Intervention 63.5 ± 11, Control 61.5 ± 11 SARA: Intervention 12.2 ± 3.2, Control 11 ± 3.7	Multi	Intensive Physical Rehabilitation including general conditioning, ROM ex., muscle strengthening, balance tr., walking, stair climbing, OT for improving ADLs.	PT 1 h/day, OT 1 hour/day, 5 day/week, 4 weeks	No intervention	SARA, FIM, Gait speed (m/s), FAC, Falls (per week)
2	Kaut et al. (36)	RCT	SCA1 (*n* = 7), SCA2 (*n* = 1), SCA3 (*n* = 11), SCA6 (*n* = 13)	Age: Intervention 61.2 ± 12.3, Control 57.3 ± 12.7 SARA: Intervention 14.31 ± 5.7, Control 11.63 ± 6.2	Sigle	WBV	5 stimulus trains of 60 s/day, 4 sequent days	Sham-vibration	SARA, 8MW, 9HPT, INAS
3	Seco et al. (40)	RCT	FA (*n* = 16)	Age: Intervention 48.2 ± 3.9, Control 56.4 ± 4.1 100% Wheelchair user No numerical data for ICARS has been reported.	Multi	PT (balance, coordination, weight tr. etc) 60 min/day	5 year, 60 minuets/session, 3 times/week,	No intervention	ICARS, FIM, SF36
4	Chang et al. (34)	RCT	SCA (*n* = 20)	Age: Intervention 48.1 ± 5.47, Control 49.7 ± 7.57 Intervention13.5 ± 9.81 (numerical data in control was not reported)	Sigle	Home-based Cycling regimen	15 min/day, 3 day/week, 4 weeks	No intervention	ICARS
5	Bunn et al. (33)	RCT	SCA6 (*n* = 12)	Age: Intervention 60.2 ± 10.5, Control 58.3 ± 14.5 SARA: Intervention 11.8 ± 6.7, Control 12.3 ± 8.5	Sigle	Home-based balance exercises including balance control engagement under functionally relevant daily scenarios while looking at projected images (optokinetic stimuli)	15 min of training, 5 days per week	No intervention	SARA, FIM, BBS, ABC, EQ-5D, EQ-VAS
6	Milne et al. (37)	RCT	FA (*n* = 19)	Age: Intervention 37.73 ± 9.81, Control 35.94 ± 15.11 FARS: Intervention 101.3 ± 22.49, Control 90.5 ± 21.04	Multi	Outpatient rehabilitation program, including strengthening, postural control, coordination and control, functional mobility, balance training, stretching and mobilizing, and cardiovascular fitness	2–3 h, 3 times/week, 6 weeks	No intervention	BBS, FIM, FARS
7	Wang et al. (43)	RCT	SCA3 (*n* = 9)	Age: Intervention 57[44-61], Control 54[51-60] SARA: Intervention 5[3.5-10], Control 7.5[5.5-13]	Single	Exergames enhancing balance training	40 min/session, 3 sessions/week, 4 weeks	Conventional balance and coordination training (30 min)	SARA, 9HPT [more affectedside], 9HPT [less affectedside]
8	Rodriguez-Diaz et al. (39)	RCT	SCA (*n* = 38)	Age: Intervention 39.52 ± 10.72, Control 38.78 ± 10.53 SARA: Intervention 15.8 ± 9.7, Control 15.9 ± 9.4 (numerical data was not reported, and estimated from graph)	Multi	Neurorehabilitation therapy, PT: emphasizing on balance, coordination, and muscle strengthening	Total 5.5 h/weekday, (PT 4 h, OT 1 h, psychotherapy 0.5 h), 24 week	No intervention	SARA, INAS
9	Tercero-Perez et al. (41)	RCT	SCA7 (*n* = 18)	Age: Intervention (intensive) 38.6 ± 14.22, Intervention (moderate) 41.33 ± 16.17, Control 39.71 ± 18.17 SARA: Intervention (intensive) 16.4 ± 6.39, Intervention (moderate) 18.58 ± 3.64, Control 15.64 ± 5.33	Multi	Strengthening, coordination tr. Balance tr., Gait tr.	Intensive tr. Group: 2 h/day, 5 day/week, 24 weeks Moderate tr. Group: 2 h/day, 3 day/week, 24 weeks	Non-training	SARA, INAS, Barthel Index
10	Velazquez-Perez et al. (42)	RCT	SCA2 (*n* = 14)	Age: Intervention 38.33 ± 8.23, Control 38.64 ± 10.34 SARA: Intervention 0.87 ± 0.79, Control 0.93 ± 0.85	Multi	Balance, gait, limb coordination training	4 h/day, 5 day/week, 3 weeks	Not receive rehabilitation	SARA, INAS, 9HPT (dominant hand)
11	Barbuto et al. (32)	RCT	SCA (*n* = 6), Idiopathic Ataxia (*n* = 7), MSA-C (*n* = 7)	Age: Intervention 53.8 ± 17.4, Control 46.1 ± 13.3 SARA: Intervention 9.1 ± 2.9, Control 10.35 ± 3.5	Single	Aerobic training with cycling regimen at home	30 min/session, 5 sessions/week, 4 weeks	No training (4 weeks)	SARA, Gait speed (m/s), TUG, DGI
12	Ayvat et al. (29)	RCT, cross-over	SCA (*n* = 7), MS (*n* = 13)	Age: Group1 32 [26–39.5], Control 34 [28–40] (median[IQR]) Numerical ICARS data in baseline of both group were not reported.	Single	Whole body vibration and exercise program	WBV: 4 min/day, Ex.:1 h/session, 3 session/week, 8 weeks	Only exercise program (same time to intervention group)	ICARS, BBS, TUG
13	Barbuto et al. (31)	RCT	DCA (*n* = 20)	Age: 20 to 70 (not reported in each group) SARA: Intervention (Aerobic tr.) 9.1 ± 2.9, Control (Balance tr.) 10.6 ± 3.5	Single	Aerobic training with cycling regimen at home	30 min/session, 5 sessions/week, 4 weeks	Balance training at home (30 min./session, 5 sessions/week, 4 weeks), Contents and difficulty were adjusted by physiotherapist	SARA, Gait speed (m/s), TUG, DGI
14	Ozvar et al. (38)	RCT, Crossover	SCA (*n* = 13), MS (*n* = 8)	Age: 18–50 (not reported in each group) SARA or ICARS were not reported.	Single	WBV	10 min/session, only Single session	local vibration (LV)	Gait speed (m/s)
15	Jabri et al. (35)	RCT, cross-over	SCA1 (*n* = 3), SCA2 (*n* = 3), FA (*n* = 1), Niemann–Pick C (*n* = 2), ARCA1 (*n* = 1)	Age: Intervention 46 ± 13, Control 48 ± 13 SARA: Intervention 7.17 ± 0.76, Control 5.83 ± 1.61	Single	Home-based coordinative training WITH vibrotactile Sensory Augmentation at home	30 min/session, 5 sessions/week, 6 weeks	Home-based coordinative training WITHOUT vibrotactile Sensory Augmentation	SARA, TUG, mCTSIB, DGI
16	Winser et al. (45)	RCT	SCA1 (*n* = 4), SCA2 (*n* = 1), SCA3 (*n* = 9), SCA6 (*n* = 3), undetected SCA (*n* = 2)	Age: Intervention 48.67 ± 11.3, Control 46.89 ± 12.56 SARA: Intervention 9.58 ± 3.63, Control 10.5 ± 3.99	Single	Tai-Chi as balance tr.	60 min./session, 3 session/week, 12 weeks	Usual care (did not receive Tai-Chi)	BBS, SARA, EQ-VAS
17	Barbuto et al. (30)	RCT	MSA-C (*n* = 6), SCA (*n* = 10), idiopathic DCA (*n* = 20)	Age: Intervention (Aerobic tr.) 54.9 ± 16.4, Control (Balance tr.) 51.1 ± 13.3 SARA: Intervention (Aerobic tr.) 11.7 ± 5.5, Control (Balance tr.) 11.3 ± 3.7	Single	Aerobic training at home	30 min/session, 5 sessions/week, 6 months	Balance training at home	SARA, Gait speed (m/s), TUG, DGI
18	Winser et al. (44)	RCT	SCA1 (*n* = 2), SCA3 (*n* = 15), SCA11 (*n* = 2), Post-infectious cerebellar degeneration (*n* = 2), Unknown cause for ataxia (*n* = 11)	Age: Intervention 50.2 ± 14.41, Control 46 ± 14.05 SARA: no reported numerical value in baseline of both group	Single	Dual-task training (balance training with cognitive task)	60 min/session, 3 session/week, 4 weeks	Single-task training (conventional balance, coordination, and cognition training delivered separately; active control group)	SARA, BBS, EQ-VAS

RCT, randomized control trial; SCA, spinocerebellar ataxia; DCA, degenerative cerebellar ataxia; FA, Friedreich's ataxia; MSA-C, multiple system atrophy-cerebellar subtype; SARA, scale for assessment and rating of ataxia; IQR, interquartile range; PT, physical therapy; OT, occupational therapy; WBV, whole body vibration; INAS, Inventory of Non-Ataxia Signs; 9HPT, 9 hole peg test; TUG, timed up and go test; DGI, dynamic gait index; mCTSTS, modified clinical test sensory interaction and balance; ICARS, International Cooperative Ataxia Rating Scale; BBS, Berg Balance Scale; EQ-VAS, EuroQol visual analog scale.

The following additional outcomes other than the primary or second outcomes were extracted: ABC (46), FAC (47), 8MWT (48), TUG (49), fall frequency, FARS (50), EQ-5D (51), 9HPT (52), modified Clinical Test of Sensory Interaction in Balance (mCTSIB) (53), BI (54), and Short form 36 (55) (Supplementary Figures 8–26).

### 3.3 RoB in studies

**Figure 2:**
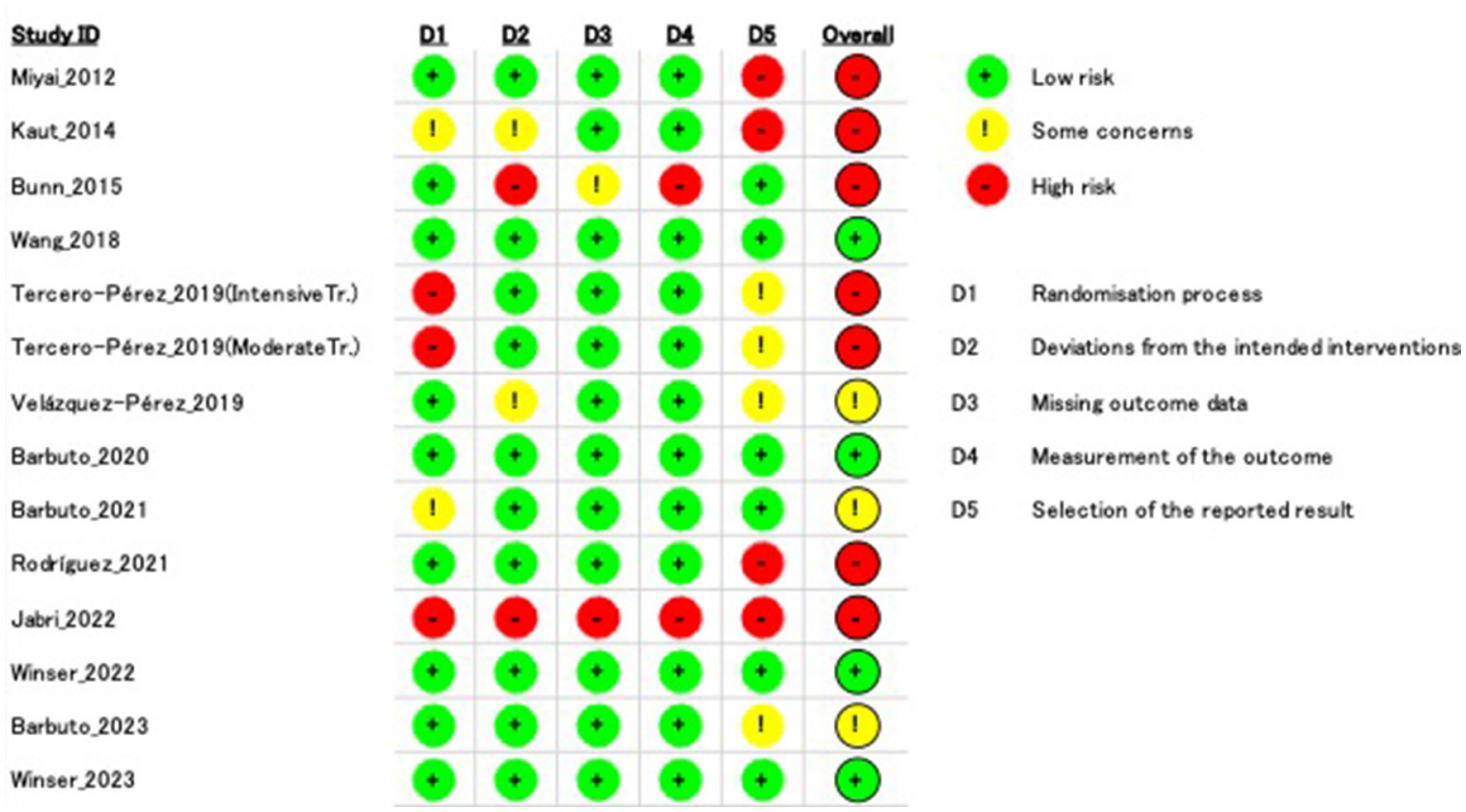
Risk of bias (RoB) based on the scale for assessment and rating of ataxia (SARA). “–” indicates “high RoB,” “!” indicate “some concerns,” and “+” indicates “low RoB.”

**Figure 3:**
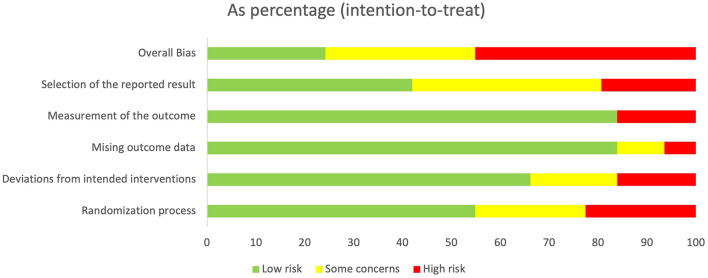
Percentage of number of studies about Risk of Bias (RoB) in intention to treat. Horizontal scale indicates percentage of number of studies. Vertical categories indicate the domain of RoB.

The agreement rate between reviewers for all outcomes across the studies, requiring the support of a third reviewer, was 12.9% (8/62), with full consensus ultimately achieved. Figure 2 and Supplementary Figures 1–7 indicate the RoB for SARA, ICARS, INAS, FIM, DGI, gait speed, BBS, and EQ-VAS. Figure 3 shows the percentages of studies in the six domains and overall bias. In terms of overall RoB, ~40% of the studies were classified as “high risk,” and approximately 20% were classified as “low risk.”

### 3.4 Results of syntheses

In case data on the SD_change_ of MD (pre-post) in the original report were insufficient, we requested the data from the corresponding authors. The authors of two studies (34, 36) provided the requested data. We received no responses to our data request for six studies (29, 39–43).

Tables 2, 3 indicate the GRADE quality of the evidence for the primary (Table 1) and secondary (Table 2) outcomes, and the RoBs in these outcomes were “serious” or “very serious.”

**Table 2:** Evidence table of SARA as primary outcome.

**Certainty assessment**							**No. of patients**		**Effect**		**Certainty**	**Importance**
**No. of studies**	**Study design**	**Risk of bias**	**Inconsistency**	**Indirectness**	**Imprecision**	**Other considerations**	**Physiotherapy**	**Control**	**Relative (95% CI)**	**Absolute (95% CI)**		
**Scale for assessment and rating of ataxia (scale from: 0 to 40)**												
14	Randomized trials	Serious^a^	Very serious^b^	Not serious	Serious^c^	Publication bias strongly suspected^d^	158	157	-	MD **1.41 point lower** (2.16 lower to 0.66 lower)	⊕◯◯◯ Very low	CRITICAL

CI, confidence interval; MD, mean difference;

a 50% of studies were judged as high risk of bias;

b I^2^ > 80%;

c The sample size was too small (<400);

d publication bias was estimated by funnel plots. The bold texts indicate important effect size.

**Table 3:** Evidence table of secondary outcomes.

	**Certainty assessment**							**No. of patients**		**Effect**		**Certainty**	**Importance**
**Outcome**	**No. of studies**	**Study design**	**Risk of bias**	**Inconsistency**	**Indirectness**	**Imprecision**	**Other considerations**	**Physiotherapy**	**Control**	**Relative (95% CI)**	**Absolute (95% CI)**		
ICARS	3	Randomized trials	Very serious°	Serious^e^	Not serious	Extremely serious^c,e^	None	10	10	-	MD **1.1 point lower** (1.77 lower to 0.43 lower)	⊕◯◯◯ Very low	IMPORTANT
INAS	-	-	-	-	-	-	-	-	-	-	-	-	IMPORTANT
FIM	3	Randomized trials	Serious^a^	Not serious	Not serious	Serious^c^	Publication bias strongly suspected^d^	36	36	-	MD **1.39 point higher** (0.59 higher to 2.19 higher)	⊕◯◯◯ Very low	IMPORTANT
DGI	4	Randomized trials	Very serious^f^	Serious^g,h^	Not serious	Extremely serious^c,i^	Publication bias strongly suspected^d^	40	37	-	MD **0.07 point higher** (1.67 lower to 1.81 higher)	⊕◯◯◯ Very low	IMPORTANT
Gait speed	6	Randomized trials	Very serious^j^	Serious^b^	Serious^k^	Serious^c,i^	Publication bias strongly suspected^d^	84	80	-	MD **0.04 m/s higher** (0.03 lower to 0.11 higher)	⊕◯◯◯ Very low	IMPORTANT
BBS	4	Randomized trials	Serious^a^	Very serious^l,m^	Not serious	Very serious^m^	None	25	26	-	MD **3.95 point higher** (1.94 lower to 9.84 higher)	⊕◯◯◯ Very low	IMPORTANT
EQ-VAS	3	Randomized trials	Serious^n^	Not serious	Not serious	Serious^c^	None	30	32	-	MD **6.84 % lower** (14.44 lower to 0.76 higher)	⊕⊕◯◯ Low	IMPORTANT

CI, confidence interval; MD, mean difference; ^a^50% of studies were judged as high risk of bias, ^b^I^2^ > 80%; ^c^The sample size was too small (<400); ^d^publication bias was estimated by funnel plots; ^e^Single publication; ^f^25% of study was judged as High risk of bias, and 75% of studies were judged as Some concerns; ^g^effect direction was inconsistent; ^h^I^2^ > 90%; ^i^divergence in 95% CI; ^j^4/6 were Some concerns and 1/6 study was High RoB; ^k^one publication (39) included participant with MS; ^l^I^2^ > 40%; ^m^The MD and SD were not reported in 2 studies (30, 34); ^n^1/3 study was judged as High RoB; ^o^2/3 studies were judged as High RoB. The bold texts indicate important effect size.

**Figure 4:**
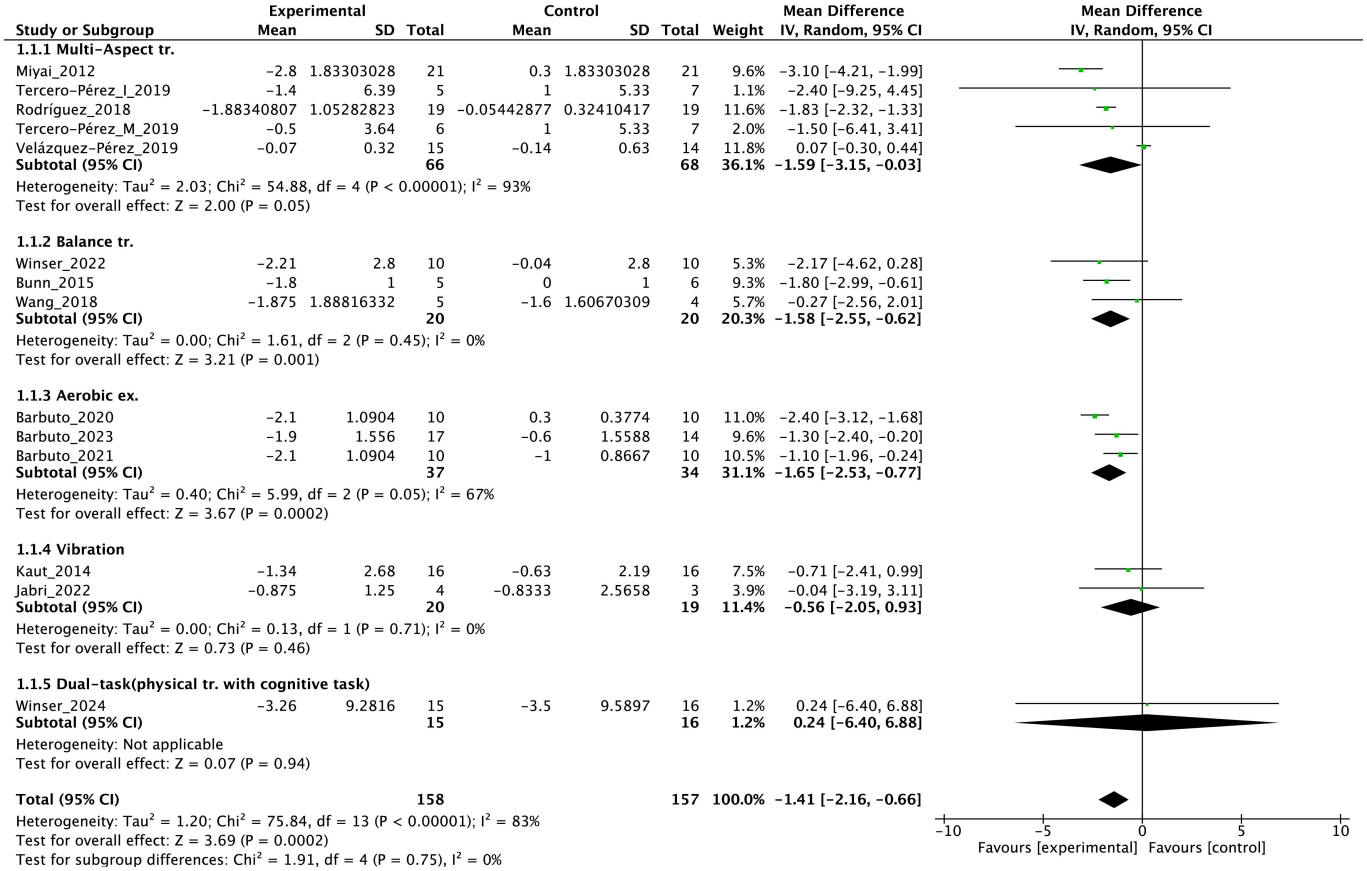
Forest plot analysis based on the scale for the assessment and rating of ataxia (SARA) as primary outcome.

**Figure 5:**
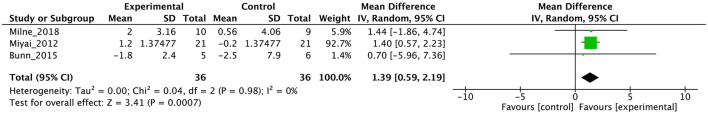
Forest plot analysis based on the functional independence measure (FIM) as one of the secondary outcomes.

**Figure 6:**
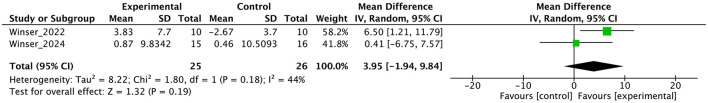
Forest plot analysis based on the Berg balance scale (BBS) as one of the secondary outcomes.

**Figure 7:**
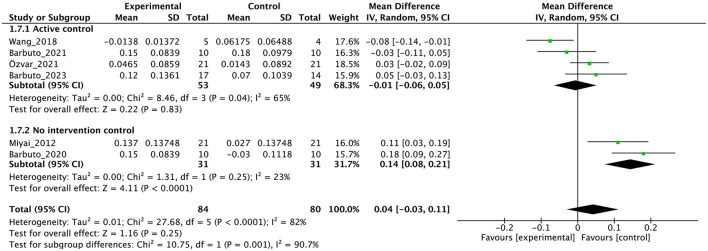
Forest plot analysis based on gait speed as one of the secondary outcomes.

**Figure 8:**
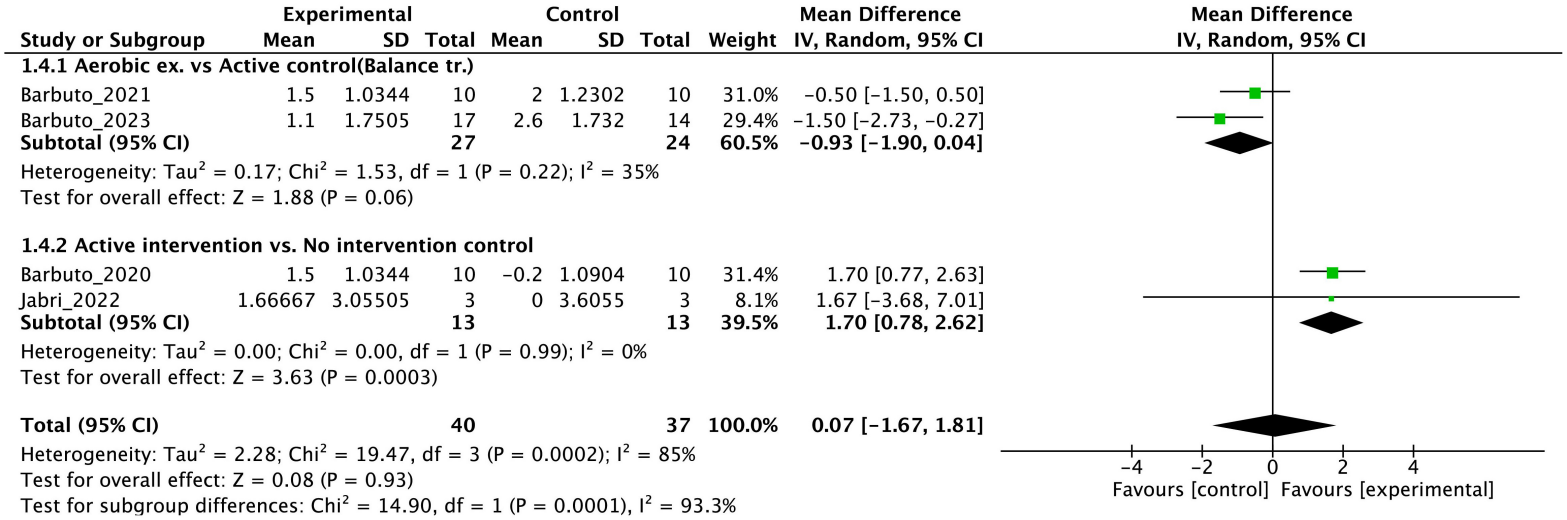
Forest plot analysis based on the Dynamic gait index (DGI) as one of the secondary outcomes.

**Figure 9:**
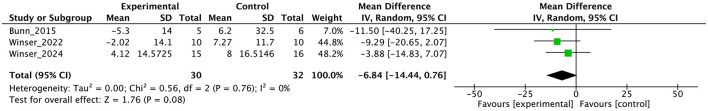
Forest plot analysis based on the Euro quality of life visual analog scale (EQ-VAS) as one of the secondary outcomes.

Individual studies and their effect sizes are shown in Figures 4–9 as forest plots. Overall, physiotherapy had a beneficial effect on SARA (MD = −1.41, 95% CI [−2.16 to −0.66], z = 3.69, *p* = 0.0002). I^2^, a statistic that indicates the level of heterogeneity (56) of the overall effect of PT on SARA, was >80%. Due to the high heterogeneity of the primary outcome, we performed subgroup analyses to explore these factors. We divided the interventions into five subgroups: (1) multi-aspect physiotherapy (MD = −1.59, 95% CI [−3.15 to −0.03], z = 2.0, p = 0.05), (2) balance training (MD = −1.58, 95% CI [−2.55 to −0.62], z = 3.21, p = 0.001), (3) aerobic exercise using cycling regimen (MD = −1.65, 95% CI [−2.53 to −0.77], z = 3.67, p = 0.0002), (4) vibration (MD = −0.56, 95% CI [−2.05 to 0.93], z = 0.73, p = 0.46), and (5) dual-task training (physical training with cognitive task) (MD = 0.24, 95% CI [−6.4 to 6.88], z = 0.07, p = 0.94). No significant difference was observed among the five subgroups (χ^2^ = 1.91, df = 4, p = 0.75, I^2^ = 0%).

In terms of secondary outcomes, a significant overall effect was observed on ICARS using single-study data (MD = −1.1, 95% CI [−1.77 to −0.43], z = 3.23, p = 0.001) and FIM (MD = 1.39, 95% CI [0.59 to 2.19], z = 3.41, *p* = 0.0007). In terms of gait speed (m/s), overall effect was not significant (MD = 0.04, 95% CI [−0.03 to 0.11], z = 1.16, *p* = 0.25, I^2^ = 82%). Subgroup analysis suggested a significant effect in the no-intervention control group setting (MD = 0.14, 95% CI [0.08 to 0.21], z = 4.11, *p* < 0.0001) but not in the active control group setting (MD = −0.01, 95% CI [−0.06 to 0.05], z = 0.22, *p* = 0.83).

### 3.5 Reporting biases

**Figure 10:**
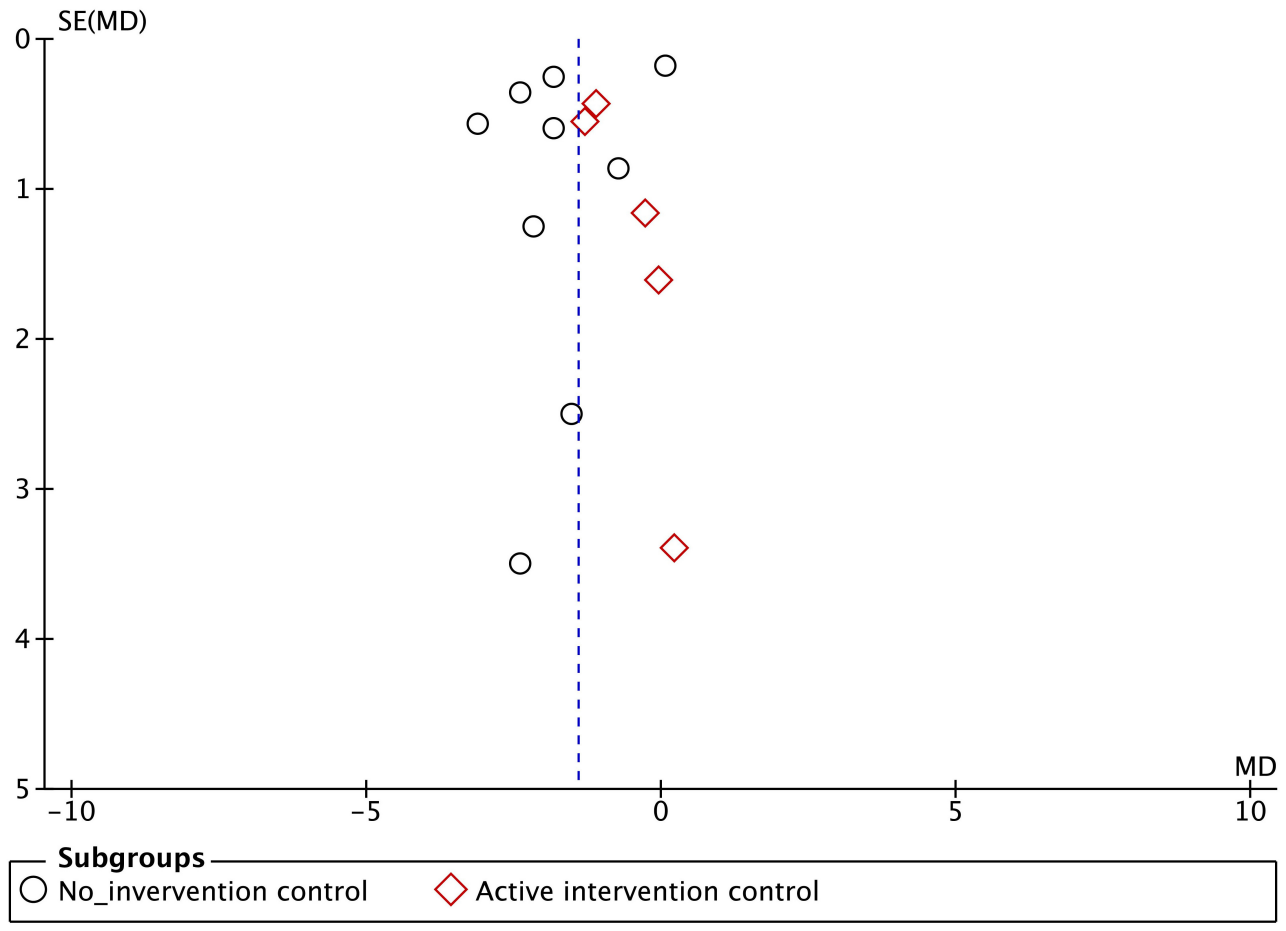
Funnel plot of the scale for assessment and rating of ataxia (SARA). SE, standard error; MD, mean difference.

Publication bias was suspected based on funnel plots for SARA all over the result (Figure 10). However, when judging each subgroup, the number of reports for each is <10, so it is difficult to fully estimate publication bias.

The funnel plot for SARA all over the result shows an asymmetrical distribution, with fewer data points in the lower left and lower right regions (Figure 10). This pattern suggests a potential risk of publication bias, as smaller studies with non-significant or unfavorable results may be underreported or unpublished. This asymmetry may influence the overall interpretation of the results, particularly when combined with the limitations of subgroup analyses the number of reports for each is <10. As a result, while the evidence synthesis incorporates these findings, the certainty of evidence should be interpreted with caution.

### 3.6 Certainty of evidence

The GRADE quality of the evidence of primary outcome SARA was judged as “very low” (Table 2). The RoB was “serious,” inconsistency was “very serious” with I^2^ >80%, imprecision was “serious” owing to the small sample size (*n* < 400), and publication bias was strongly suspected as the reason for the obvious asymmetry of the funnel plot (Figure 10).

In the secondary outcomes, the result of GRADE quality is shown in Table 3. Notably, INAS data were not obtained from all authors reported in the original publication of INAS. Therefore, none of the results of GRADE were generated. In other secondary outcomes, certainty of evidence was judged as “very low” in ICARS, FIM, gait speed, DGI, and BBS and “low” in EQ-VAS.

## 4 Discussion

This study evaluated the effectiveness of physiotherapy interventions in patients with DCA. The results of this systematic review and meta-analysis demonstrated that physiotherapy significantly reduces ataxia symptoms with no adverse event, as evidenced by a notable decrease in the SARA scores. Specifically, a multi-faceted physiotherapy approach—including balance, aerobic, muscle strength, coordination, gait, and ADL training—was particularly effective in alleviating ataxia symptoms. These findings support the importance of physiotherapy in patients with DCA and suggest that such approaches may be widely adopted in future clinical practice without severe adverse events. However, this evidence should be used with caution because of various remaining concerns.

First, a high level of statistical heterogeneity (I^2^ > 80%) was observed for the primary outcome SARA scores. This heterogeneity could primarily be attributed to the broad range of interventions used. The included studies employed various designs, including multi-faceted interventions, such as muscle strength training, coordination training, gait training, and ADL training, or focused on specific types of training, such as aerobic exercise, balance training, and vibration stimulation. Each of these interventions possibly affected patients through different mechanisms, which potentially led to variability in treatment effects. Additionally, some studies used an active control group rather than a no-intervention control group. This inclusion of active controls possibly introduced effects from non-specific factors, such as placebo or learning effects, contributing to the variability in outcomes. Furthermore, the variation in intervention duration among studies possibly played a role in increasing heterogeneity. Some studies implemented interventions over a few weeks, whereas others extended over several months, which could impact the outcomes differently. Another possible factor is severity of disease and symptoms, and As highlighted by Reetz et al. (57), SARA items related to trunk and lower limb functions may exhibit ceiling effects after the loss of walking ability, reducing their sensitivity to detect disease progression. This limitation could have influenced the observed variability in treatment effects in our analysis, particularly in non-ambulatory patients. Finaly, the effects may differ depending on the type of disease (58, 59), and this may have affected the results. These factors combined contributed to the high statistical heterogeneity observed, making the aggregated results difficult to generalize. When interpreting the findings of this study, we should consider these sources of heterogeneity, carefully evaluating the specific characteristics of each intervention, the type of control groups used, and the influence of intervention duration on the outcomes.

Different from previous systematic reviews (5, 60, 61), the present systematic review and meta-analysis highlighted that aerobic exercise (30–32), such as cycling regimens, can notably reduce cerebellar ataxia symptoms, as reflected in the significant improvements in SARA scores. These exercises contribute to cardiovascular fitness, which may enhance overall endurance and mobility in patients with DCA. The repetitive and rhythmic nature of aerobic activities could also promote neuroplasticity, aiding in the reorganization and adaptation of motor function (34). These findings suggest that incorporating aerobic exercise into rehabilitation programs for people with DCA could support motor aspects of health and thus contribute to a comprehensive therapeutic strategy.

Previous systematic reviews of interventions using non-invasive brain stimulation (NIBS) have shown improvements in SARA scores of over 2.5 points (62, 63), which is greater than the 1.4 points achieved through PT in this study. It is also considerably lower than SARA's minimally detectable change of 3.5 (64). While PT is a safer intervention suggested by this systematic review, this discrepancy highlights the need to explore the potential benefits of combining PT with NIBS to achieve greater therapeutic effects. A previous study examined the effects of combining repetitive transcranial magnetic stimulation (rTMS) and PT, providing a direction for future research (65), which was not included out SR because this rTMS+PT report was published after our article search for this systematic review. Additionally, whether PT offers advantages over NIBS in maintaining long-term effects should be investigated. These considerations suggest that future strategies for managing symptoms in patients with DCA should focus on integrating PT with NIBS and optimizing PT to sustain its benefits over longer periods, ultimately aiming to enhance the overall QOL for individuals with DCA.

One of the strengths of this study is its broad inclusion of a wide range of outcomes, which provides a comprehensive overview of the effects of physiotherapy on patients with DCA (5, 60, 61). However, the diversity in gait-related indicators, such as different measures of gait speed and balance, poses a challenge for integration and comparison across studies. This lack of uniformity complicates the selection of the most appropriate measures for clinical use (66). Moreover, while patient-reported outcomes (67) are increasingly recognized as important in the rehabilitation of DCA, few studies have utilized QOL-related indicators, highlighting a critical gap in current research. Addressing this gap by incorporating more QOL measures will provide a better understanding of how physiotherapy interventions impact the overall wellbeing and daily life of patients, ensuring that treatment approaches are aligned with patient-centered goals.

The results of this meta-analysis showed the limited effects of PT on secondary outcomes. The significant effects on ICARS and FIM were find, but the numbers of studies and participants were small. In gait ability estimated by gait speed and DGI, subgroup analysis showed an effect in the no-intervention control group but not in the study with active control. These findings reflect the minimal or limited effects of PT on gait. In balance ability, we cannot find significant effect on BBS, indicating the effect of PT on balance ability may be limited with note the studies select active control [usual care (45) or physiotherapy (44)]. In QOL, there was no significant effect on EQ-5D. In non-motor symptoms, we could not obtain INAS data from either study. In secondary outcomes, many studies have adopted active control, and subgroup analysis clearly shows that this has brought down the overall effect size. Therefore, it is necessary to use this evidence about secondary outcome with the understanding that it may be underestimated. Further, RoB were serious or very serious, and certainty of evidence were “low” or “very low”. Therefore, in secondary outcome, we cannot enough discuss the effectiveness or certainty of the study at all.

This study has several limitations that are common in systematic reviews and meta-analyses. One of the primary concerns was the high RoB in many of the included studies. The variation in study quality, with some studies having methodological weaknesses, affected the reliability of the overall findings. Furthermore, the certainty of the evidence was judged to be very low, raising concerns about the robustness of the conclusions drawn from this analysis. The publication bias estimated by funnel plot including 13 individual RCTs was judged as high, but the subgroup analysis involved less than 10 RCTs, so as a result, while the evidence synthesis incorporates these findings, the certainty of evidence should be interpreted with caution. These factors indicated that the results of this meta-analysis should not be directly and uniformly applied to clinical practice. Instead, clinicians must carefully consider the context of each patient's condition, the specific nature of the physiotherapy interventions, and the quality of the evidence when integrating these findings into treatment plans. Caution and clinical judgment are essential to ensure that interventions are appropriate and beneficial for individual patients with DCA.

Another notable limitation of this study was the small number of studies and the low certainty of evidence for the seven secondary outcomes selected: ICARS, FIM, gait speed, DGI, BBS, EQ-VAS, and INAS. The limited data available on these outcomes and the variability in reporting restricted the ability to draw firm conclusions about their efficacy. Thus, a core outcome set that standardizes the measurement and reporting of critical outcomes must be established in clinical trials involving patients with DCA. Establishing such a core set would enhance comparability across studies, improve the reliability of evidence synthesis, and ensure that all clinically relevant aspects of DCA are comprehensively evaluated, ultimately leading to better-targeted and more effective rehabilitation interventions.

Research into physiotherapy for DCA faces several challenges primarily because of the heterogeneity of the disease. DCA encompasses various subtypes, each with distinct pathologies and clinical presentations, leading to a wide range of symptoms and rates of progression among patients (1). This diversity complicates the design of standardized therapeutic interventions and hinders the ability to generalize findings across different DCA subtypes (68). Moreover, as a rare disease, DCA presents difficulties in recruiting sufficient sample sizes for robust clinical trials (69), which impacts the statistical power and reliability of the studies. In addition to ataxia, patients with DCA may experience cognitive impairments, spasticity, and general physical decline, which vary between individuals (70). These peripheral symptoms further complicate the assessment of physiotherapy outcomes. Thus, interventions may need to be tailored to address not only the primary ataxia symptoms but also these associated conditions. Addressing these issues requires comprehensive and adaptable research approaches that consider the full spectrum of DCA symptoms and their impact on patient health and QOL.

Another limitation of this study was that the integrated effects were based solely on data collected immediately after the intervention. The study did not account for the varying lengths of the intervention periods, which ranged from as short as 4 weeks to as long as 6 months, and even up to 5 years in some cases. Furthermore, this analysis did not investigate the duration for which the intervention effects are sustained over time. Interventions showing no immediate effect are unlikely to yield significant benefits 6 months post-intervention. However, for those interventions that demonstrated immediate positive effects, further research is needed to explore the long-term sustainability of these benefits. Future studies should focus on examining the persistence of the therapeutic effects of physiotherapy over extended periods to clarify the long-term impact of this intervention in patients with DCA.

In the context of clinical rehabilitation for progressive neurodegenerative diseases, it is essential to consider the selection of appropriate programs, the number and frequency of sessions, the optimal timing for initiation, and the customization of interventions based on the disease stage. However, this systematic review does not provide definitive answers to these questions due to the limitations of the available evidence. Given the potential benefits of physiotherapy from the early stages of the disease (42), as well as its efficacy even in cases where walking becomes difficult (40), we believe it is crucial to initiate and maintain physiotherapy as early and consistently as possible. Additionally, since the effects of physiotherapy can be sustained but may diminish over time (4), long-term intervention programs that are easy to implement and safe for home use are essential (30–32, 34). Tailoring rehabilitation programs in multiaspect with flexible intensity to address each patient's specific symptoms is a key responsibility of physiotherapists (4, 39, 41, 42), as individualized care can optimize outcomes. To provide clearer answers to these critical questions, further high-quality RCTs are urgently needed.

As mentioned above, there are many problems with physical therapy research on DCA. Nevertheless, the results of this research provide information on the factors necessary for obtaining an effect. Multi-aspect PT programs, incorporating approaches such as muscle strengthening, balance training, coordination exercises, and aerobic training, have demonstrated significant benefits for mitigating ataxia symptoms and improving overall QOL in patients with DCA. These comprehensive programs address the complex needs of patients by targeting multiple dimensions of motor function simultaneously. However, the intensity of these interventions, often exceeding 2 h per day, 5 days a week, necessitates careful planning to align with each patient's capacity and endurance, ensuring feasibility and sustainability. Tailored therapy regimens are essential to optimize outcomes while accommodating individual health conditions.

In conclusion, this systematic review and meta-analysis indicate that physiotherapy, particularly a multi-aspect approach, can significantly reduce ataxia symptoms in patients with DCA. While the findings support the incorporation of various PT interventions into patient care, the overall low certainty of evidence and high RoB necessitate careful consideration when applying these results in clinical settings. Further high-quality research is needed to strengthen the evidence base and provide clearer guidance on the most effective physiotherapy strategies for managing DCA. Nevertheless, the demonstrated safety and potential benefits of these interventions offer promising directions for improving the management and QOL of individuals with DCA.

## Data Availability

The original contributions presented in the study are included in the article/Supplementary material, further inquiries can be directed to the corresponding author.
