# Supplementary material for: Effects of physiotherapy on degenerative cerebellar ataxia: a systematic review and meta-analysis
Source: Front Neurol. 2025 Jan 10;15:1491142. doi: 10.3389/fneur.2024.1491142 (PMC11757114; doi:10.3389/fneur.2024.1491142)
Supplement: Supplementary file 2 [file Table_2.docx]

Supplementary Material

## Supplementary Figures
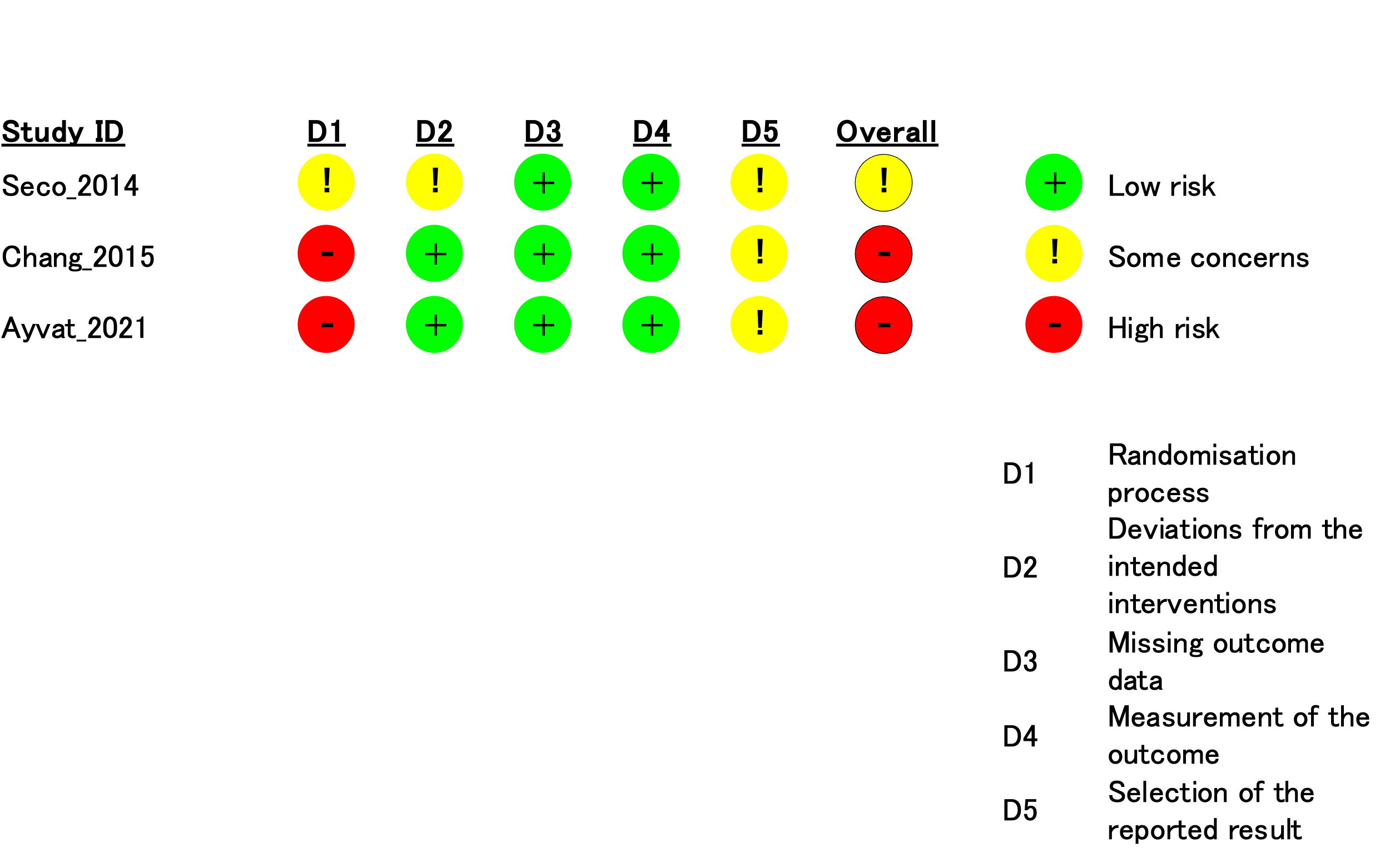


**Supplementary Figure 1.** Risk of bias (RoB) based on the scale for the International Cooperative Ataxia Rating Scale (ICARS). “–“ indicates “high RoB,” “!” indicate “some concerns,” and “+” indicates “low RoB.”


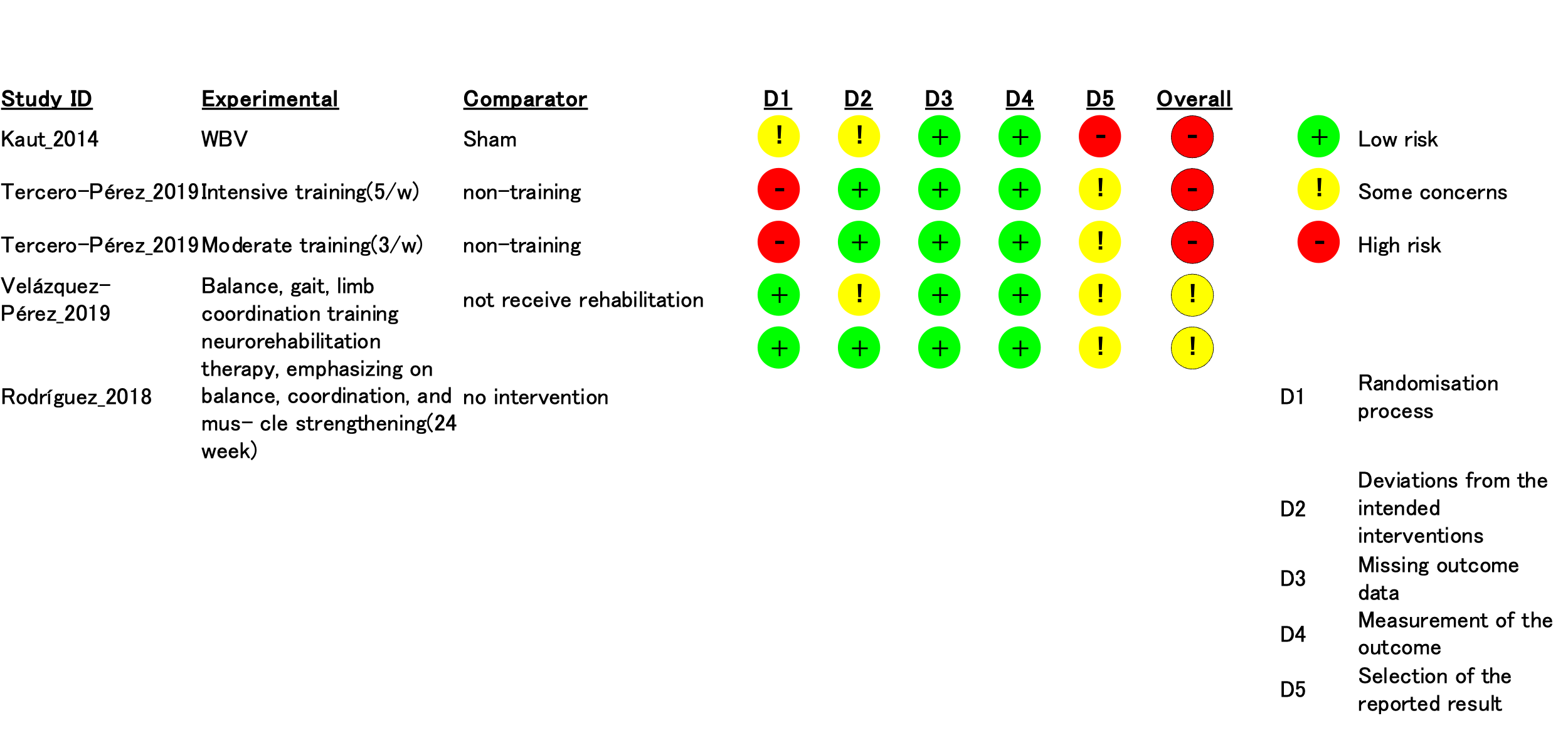


**Supplementary Figure 2.** Risk of bias (RoB) based on the Inventory of Non-Ataxia Signs (INAS). “–“ indicates “high RoB,” “!” indicate “some concerns,” and “+” indicates “low RoB.”


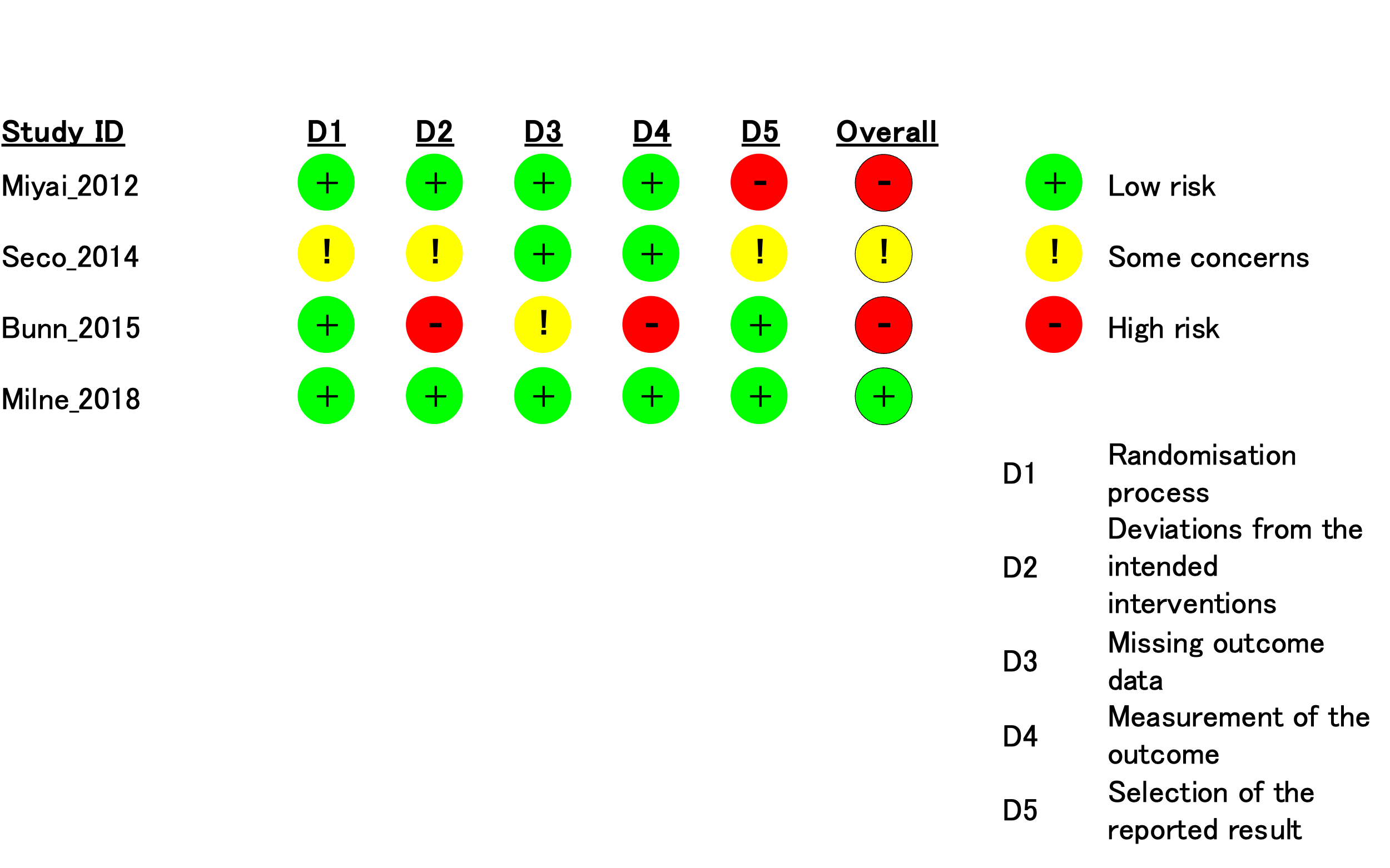


**Supplementary Figure 3.** Risk of bias (RoB) based on the functional independence measure (FIM). “–“ indicates “high RoB,” “!” indicate “some concerns,” and “+” indicates “low RoB.”


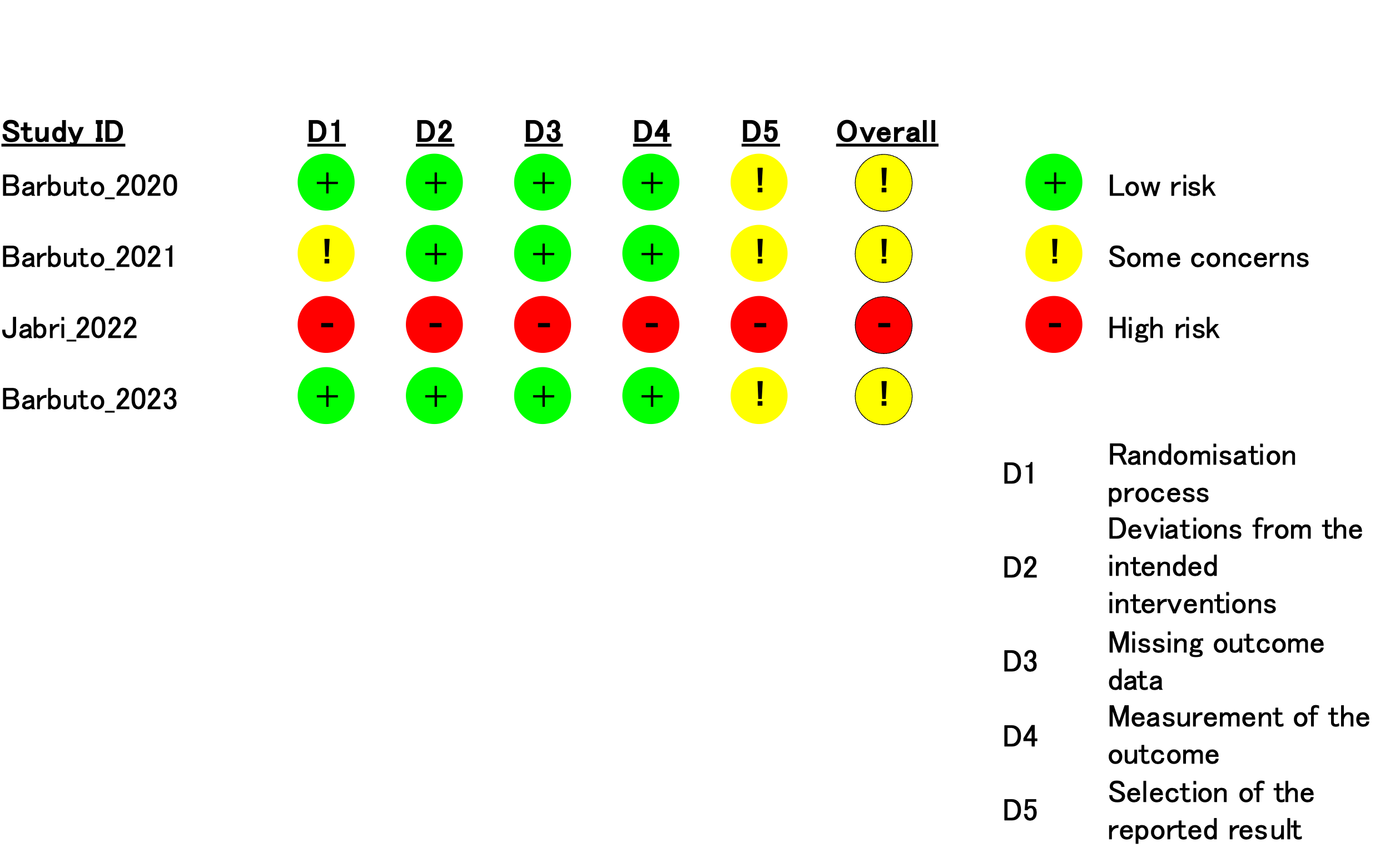


**Supplementary Figure 4.** Risk of bias (RoB) based on the dynamic gait index (DGI). “–“ indicates “high RoB,” “!” indicate “some concerns,” and “+” indicates “low RoB.”


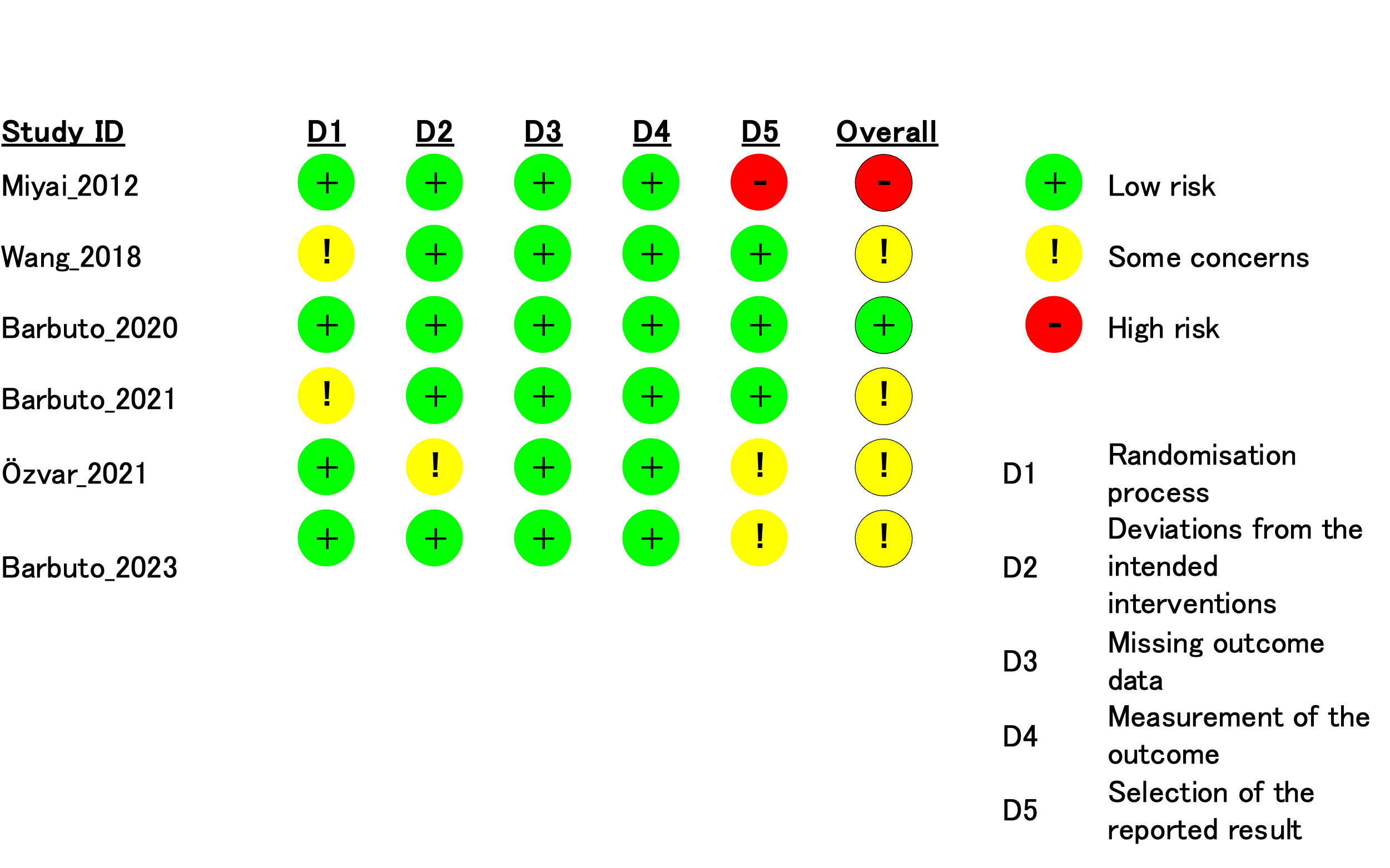


**Supplementary Figure 5.** Risk of bias (RoB) based on gait speed. “–“ indicates “high RoB,” “!” indicate “some concerns,” and “+” indicates “low RoB.”


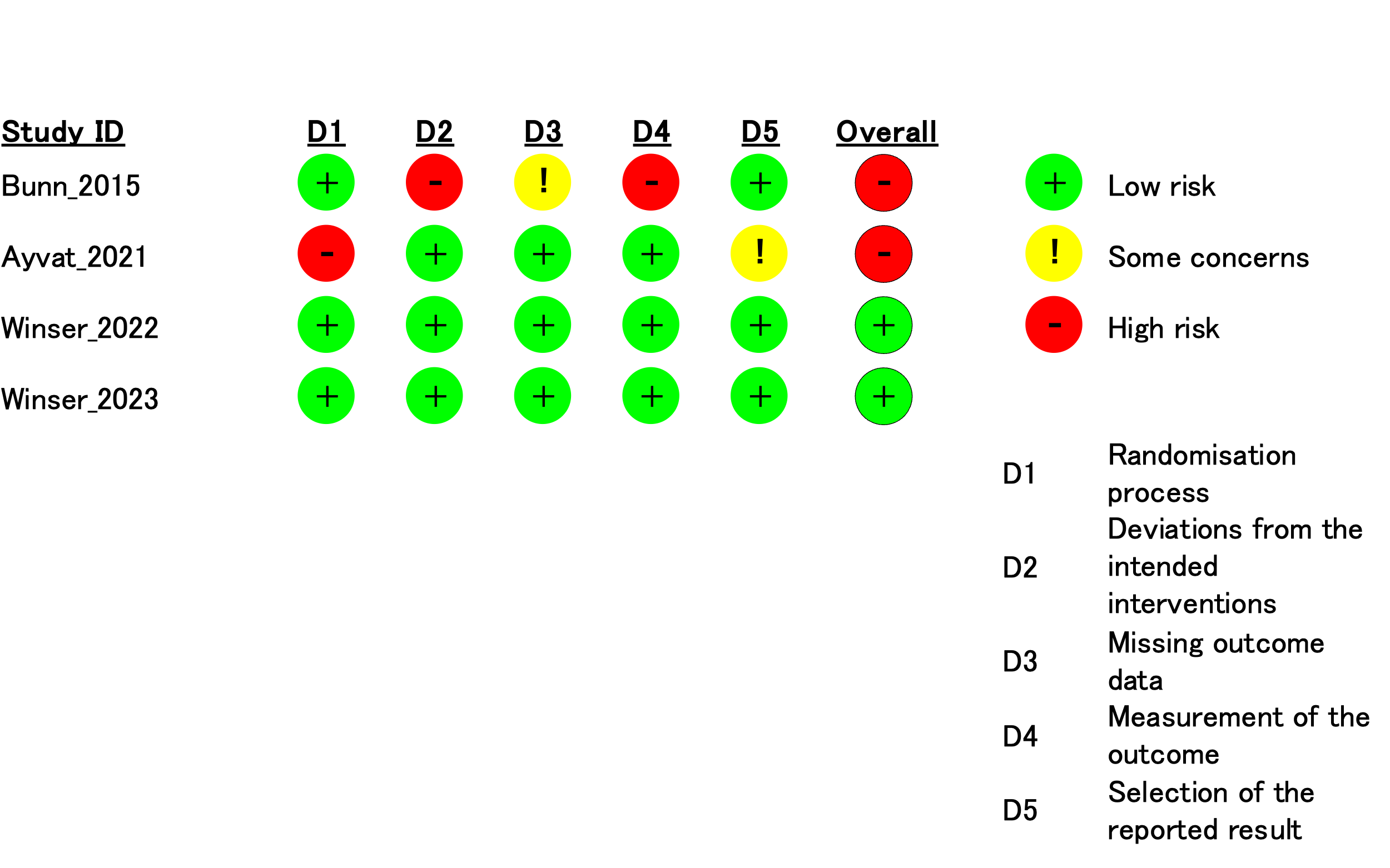


**Supplementary Figure 6.** Risk of bias (RoB) based on the Berg balance scale (BBS). “–“ indicates “high RoB,” “!” indicate “some concerns,” and “+” indicates “low RoB.”


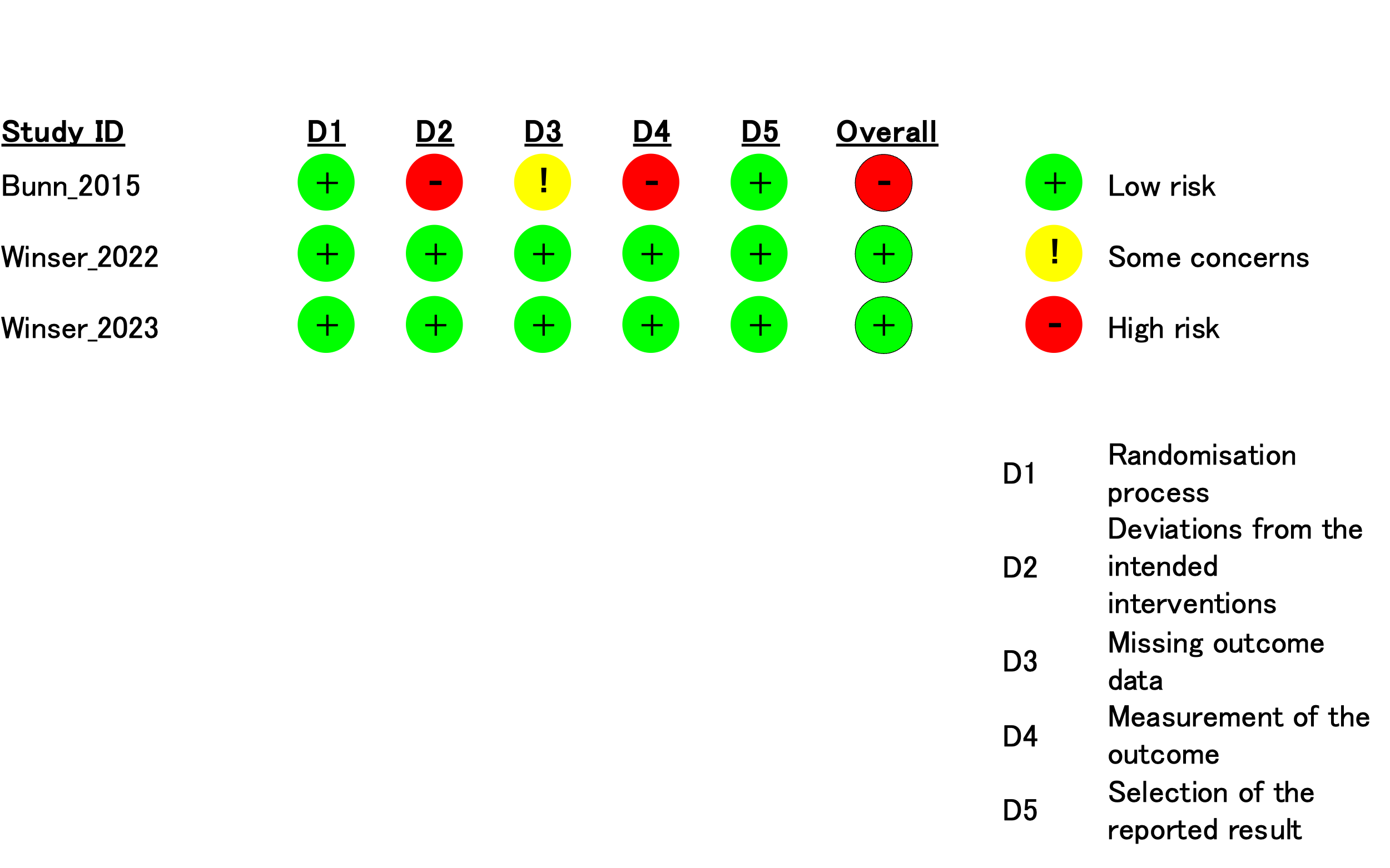


**Supplementary Figure 7.** Risk of bias (RoB) based on the Euro Quality of Life Visual Analogue Scale (EQ-VAS). “–“ indicates “high RoB,” “!” indicate “some concerns,” and “+” indicates “low RoB.”


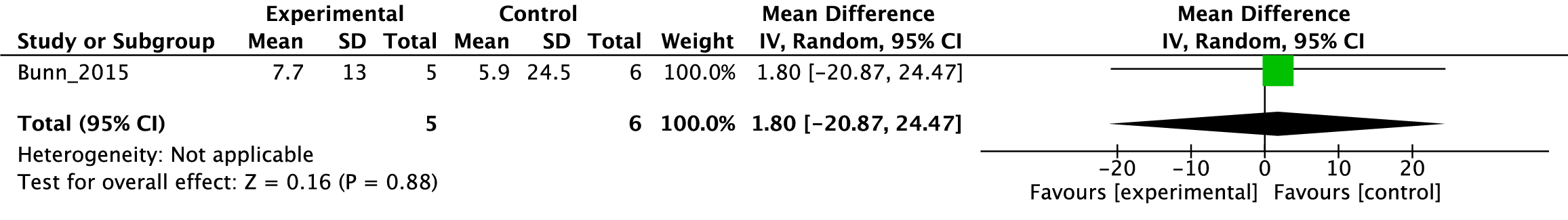


**Supplementary Figure 8.** Forest plot analysis based on the Activities of Balance Confidence questionnaire (ABC).


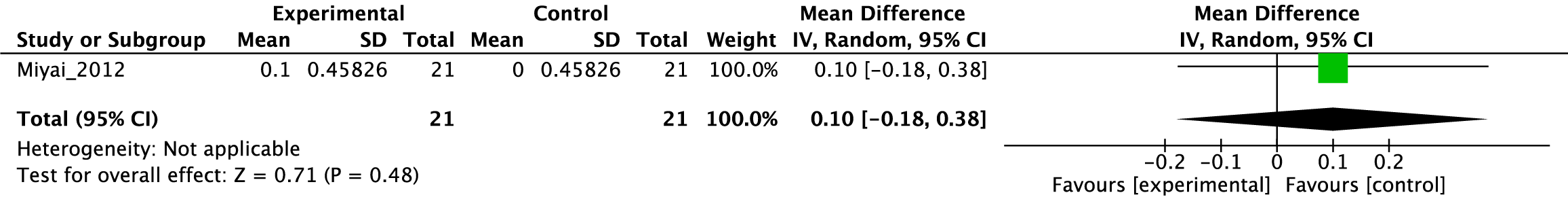


**Supplementary Figure 9.** Forest plot analysis based on the functional ambulatory capacity (FAC).


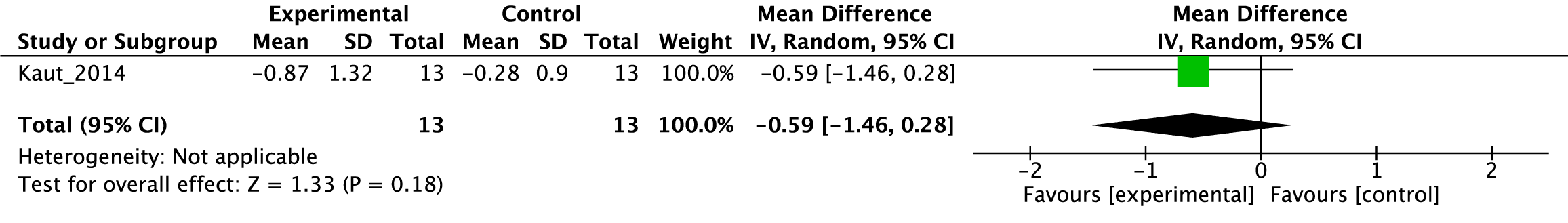


**Supplementary Figure 10.** Forest plot analysis based on 8 meter walk test (8MWT).


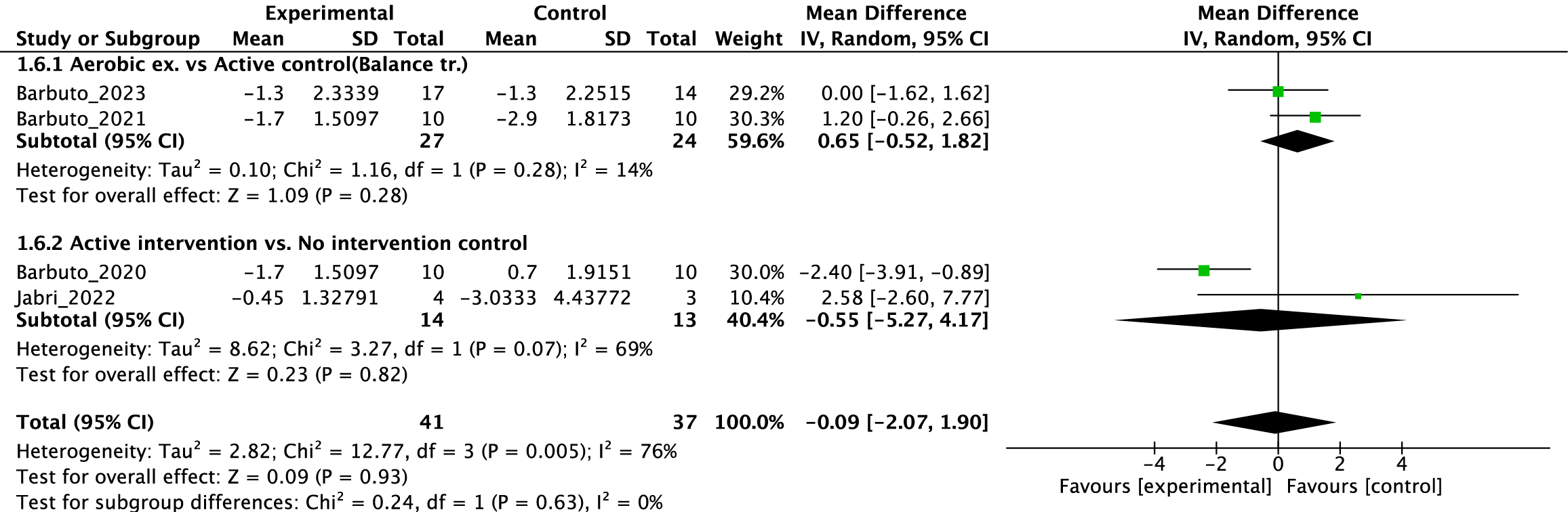


**Supplementary Figure 11.** Forest plot analysis based on the timed up and go test (TUG).


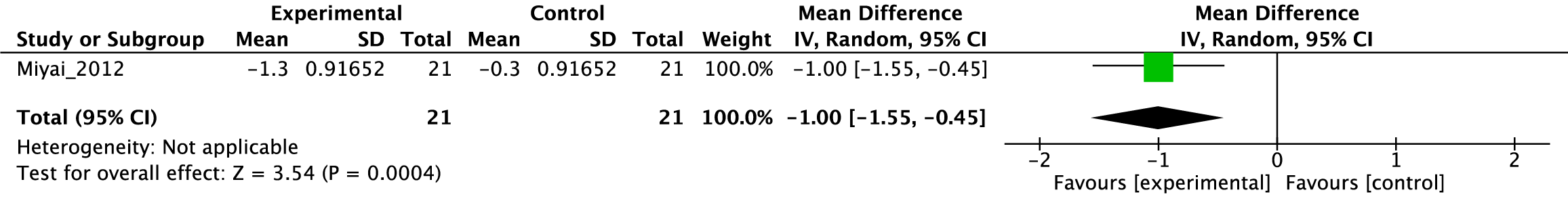


**Supplementary Figure 12.** Forest plot analysis based on fall frequency.


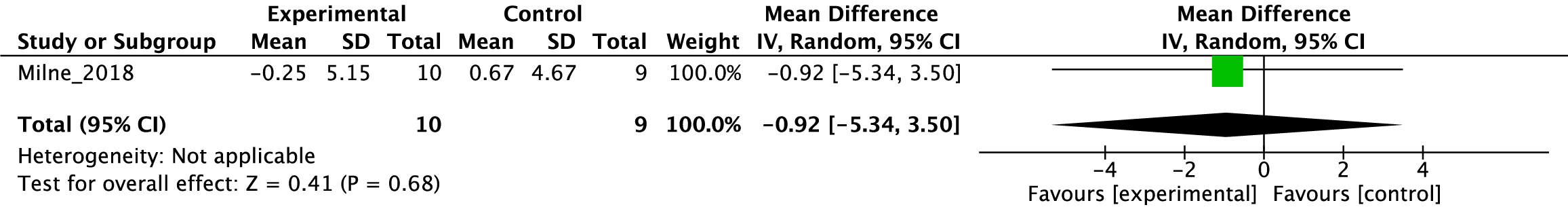


**Supplementary Figure 13.** Forest plot analysis based on the Friedreich’s Ataxia Rating Scale (FARS).


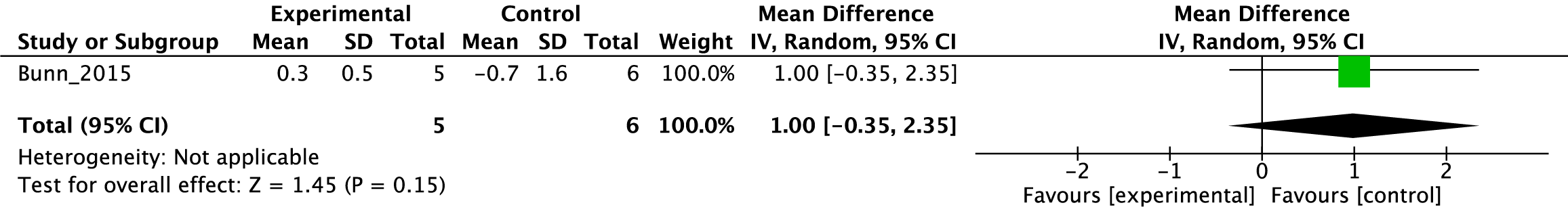


**Supplementary Figure 14.** Forest plot analysis based on the Euro quality of life 5 dimension (EQ-5D).


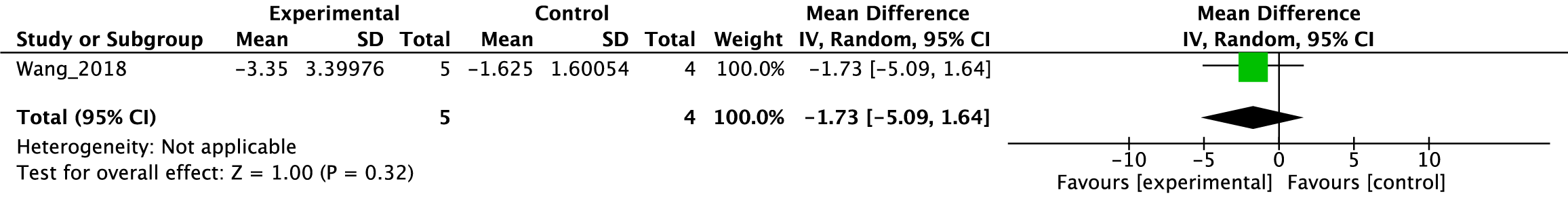


**Supplementary Figure 15.** Forest plot analysis based on the 9 hole peg test (9HPT).


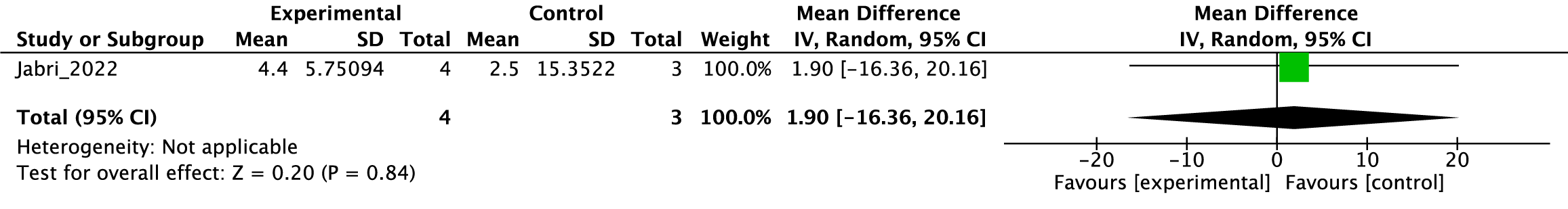


**Supplementary Figure 16.** Forest plot analysis based on the modified Clinical Test of Sensory Interaction in Balance (mCTSIB).


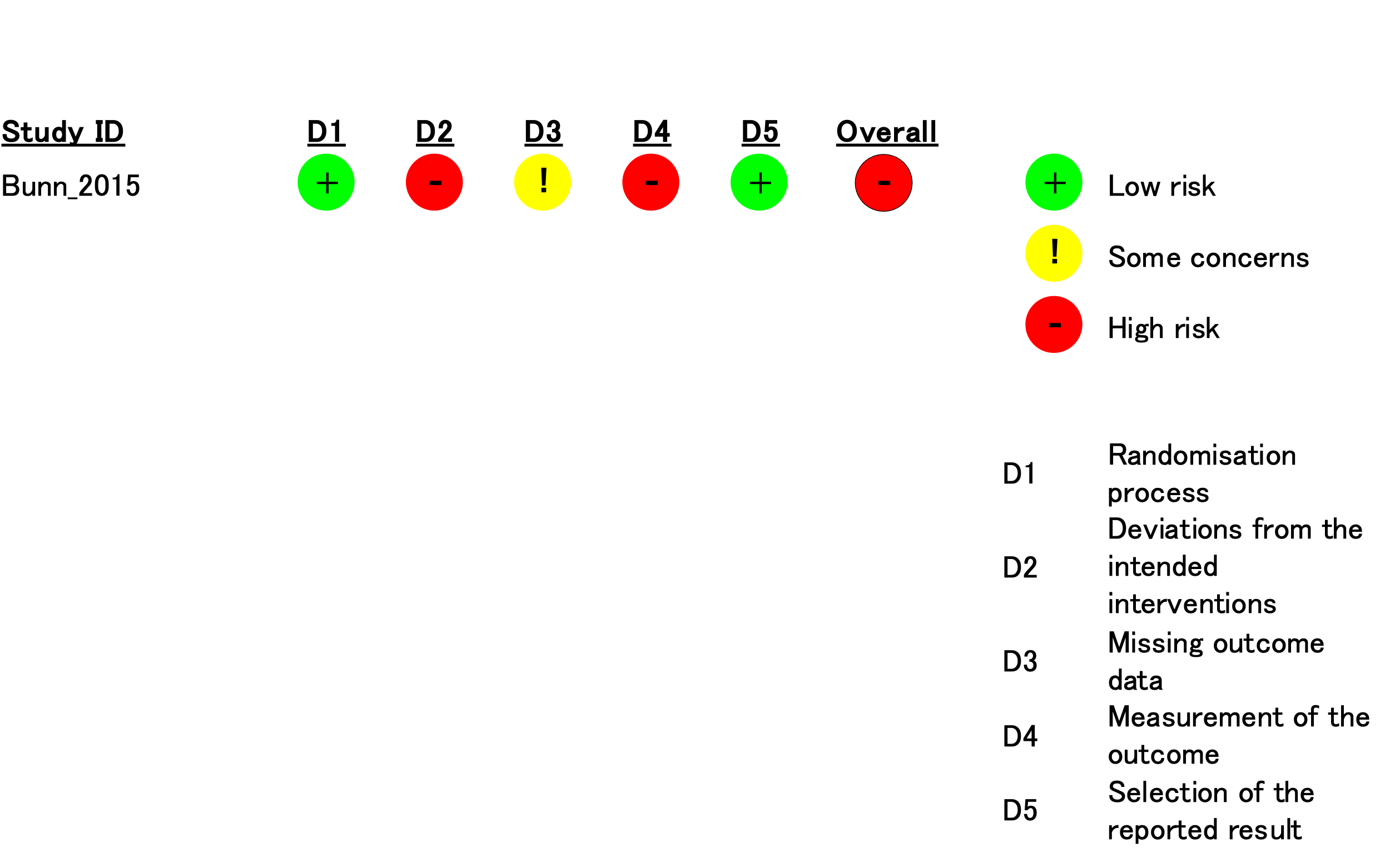


**Supplementary Figure 17.** Risk of bias (RoB) based on the Activities of Balance Confidence questionnaire (ABC). “–“ indicates “high RoB,” “!” indicate “some concerns,” and “+” indicates “low RoB.”


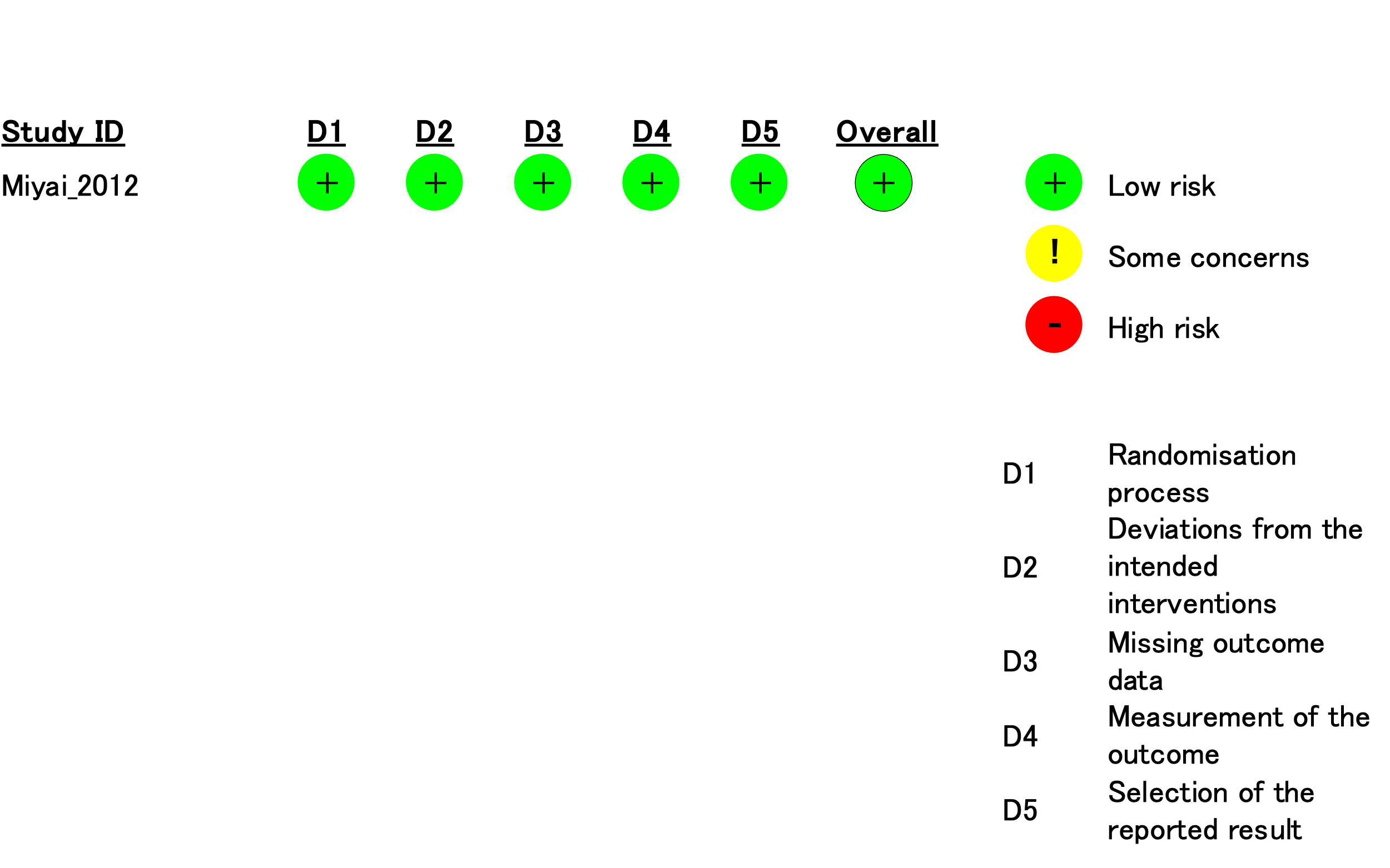


**Supplementary Figure 17.** Risk of bias (RoB) based on the functional ambulatory capacity (FAC). “–“ indicates “high RoB,” “!” indicate “some concerns,” and “+” indicates “low RoB.”


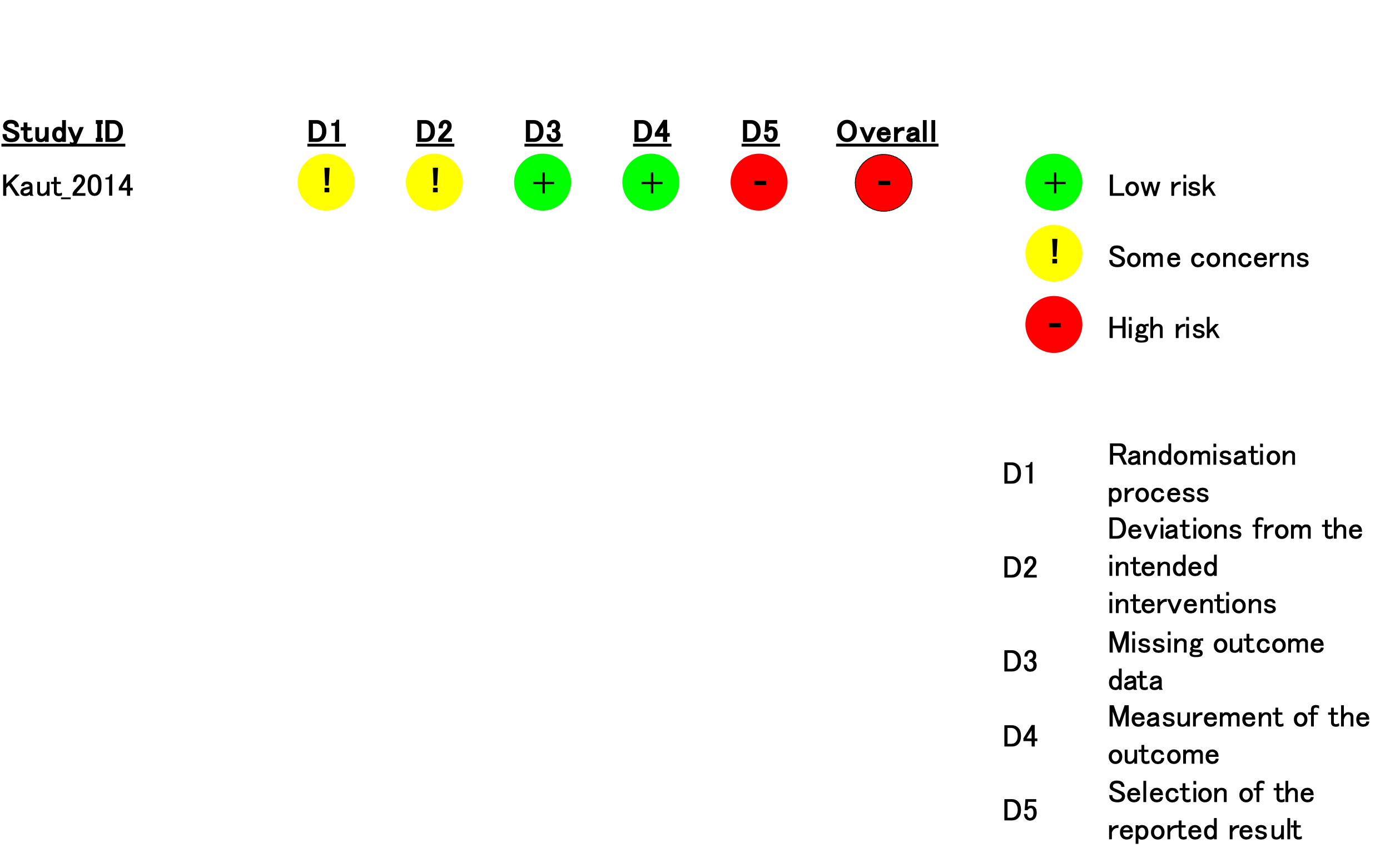


**Supplementary Figure 18.** Risk of bias (RoB) based on the 8 meter walk test (8MWT). “–“ indicates “high RoB,” “!” indicate “some concerns,” and “+” indicates “low RoB.”


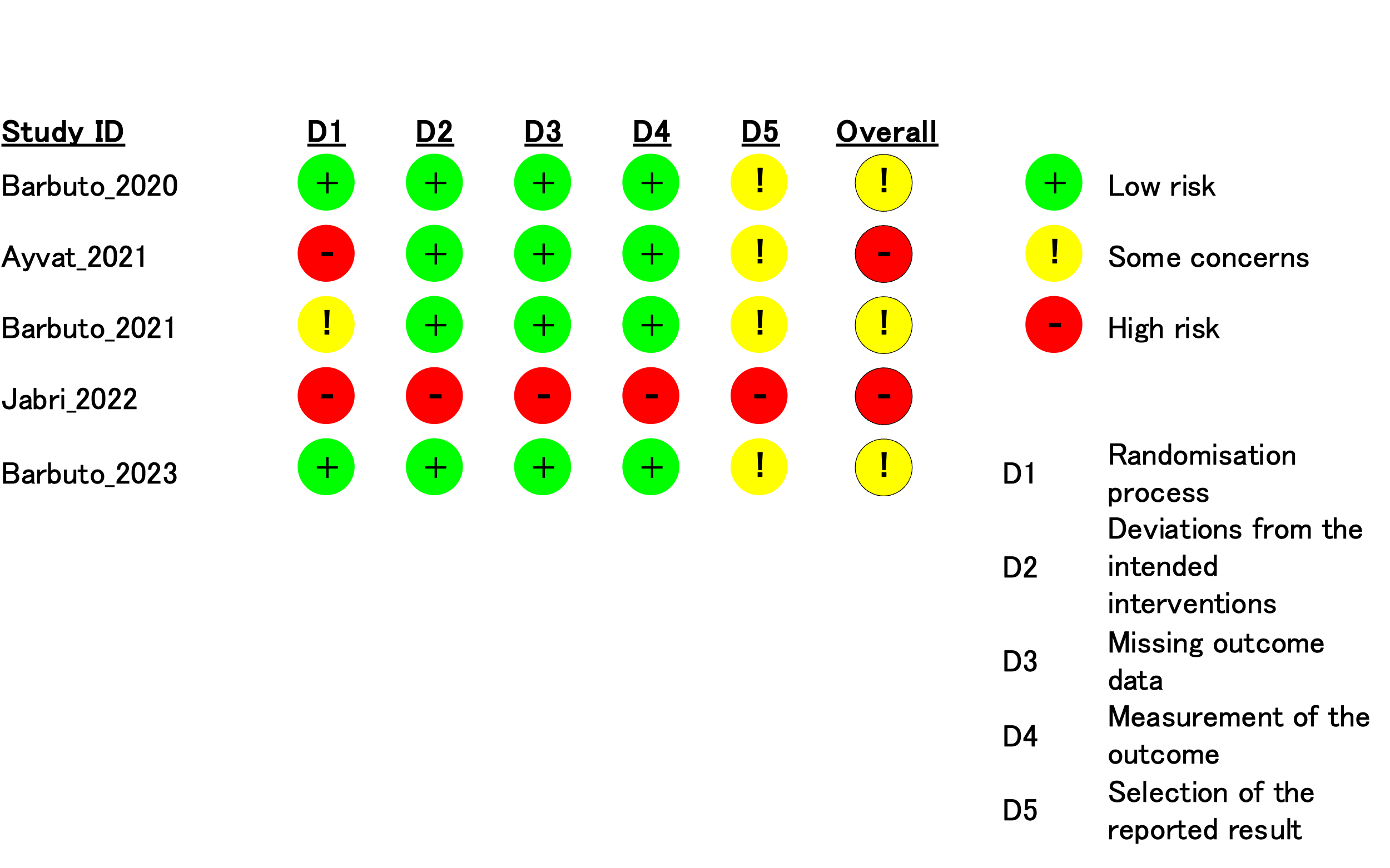


**Supplementary Figure 19.** Risk of bias (RoB) based on the timed up and go test. “–“ indicates “high RoB,” “!” indicate “some concerns,” and “+” indicates “low RoB.”


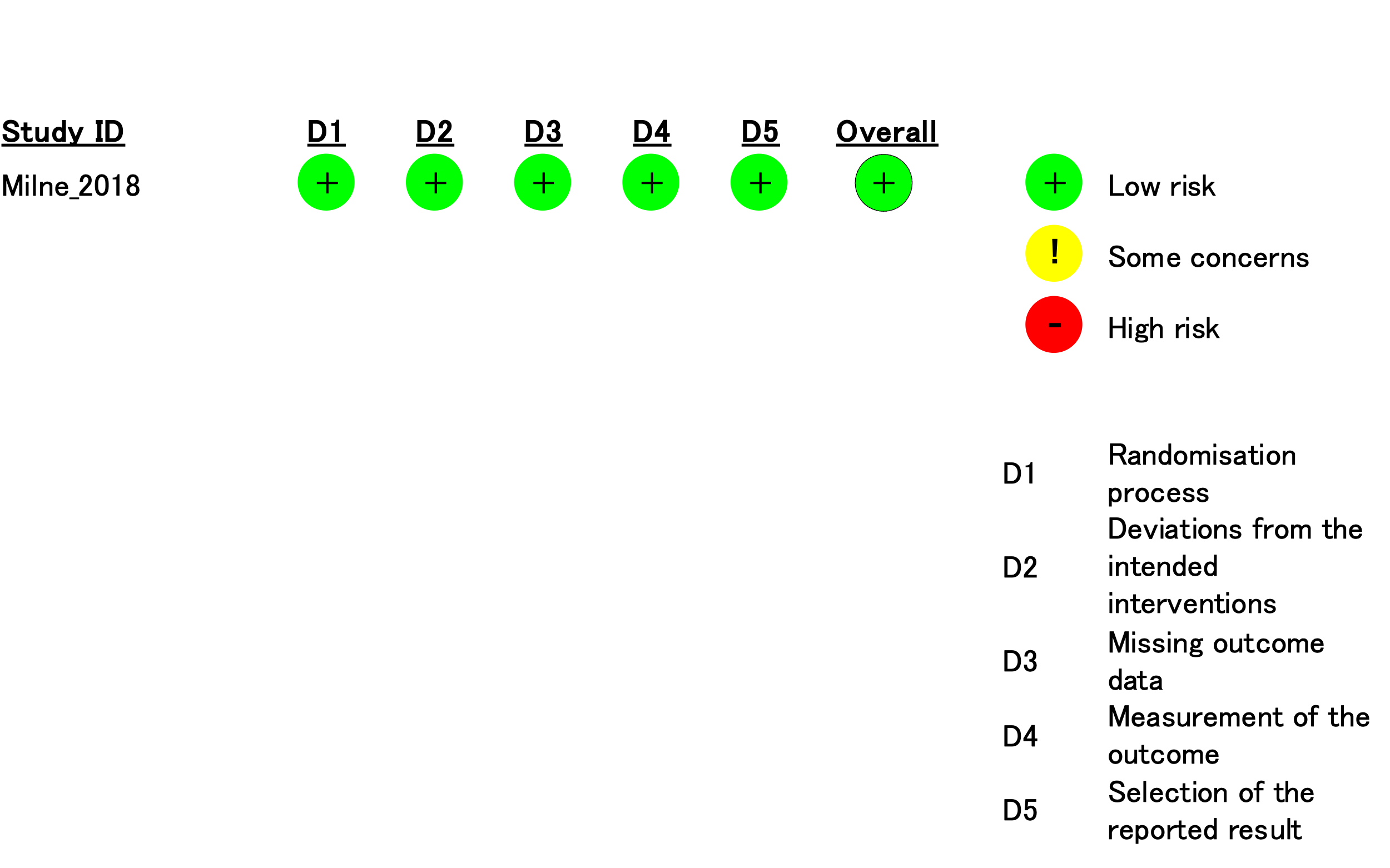


**Supplementary Figure 20.** Risk of bias (RoB) based on the Friedreich’s Ataxia Rating Scale (FARS). “–“ indicates “high RoB,” “!” indicate “some concerns,” and “+” indicates “low RoB.”


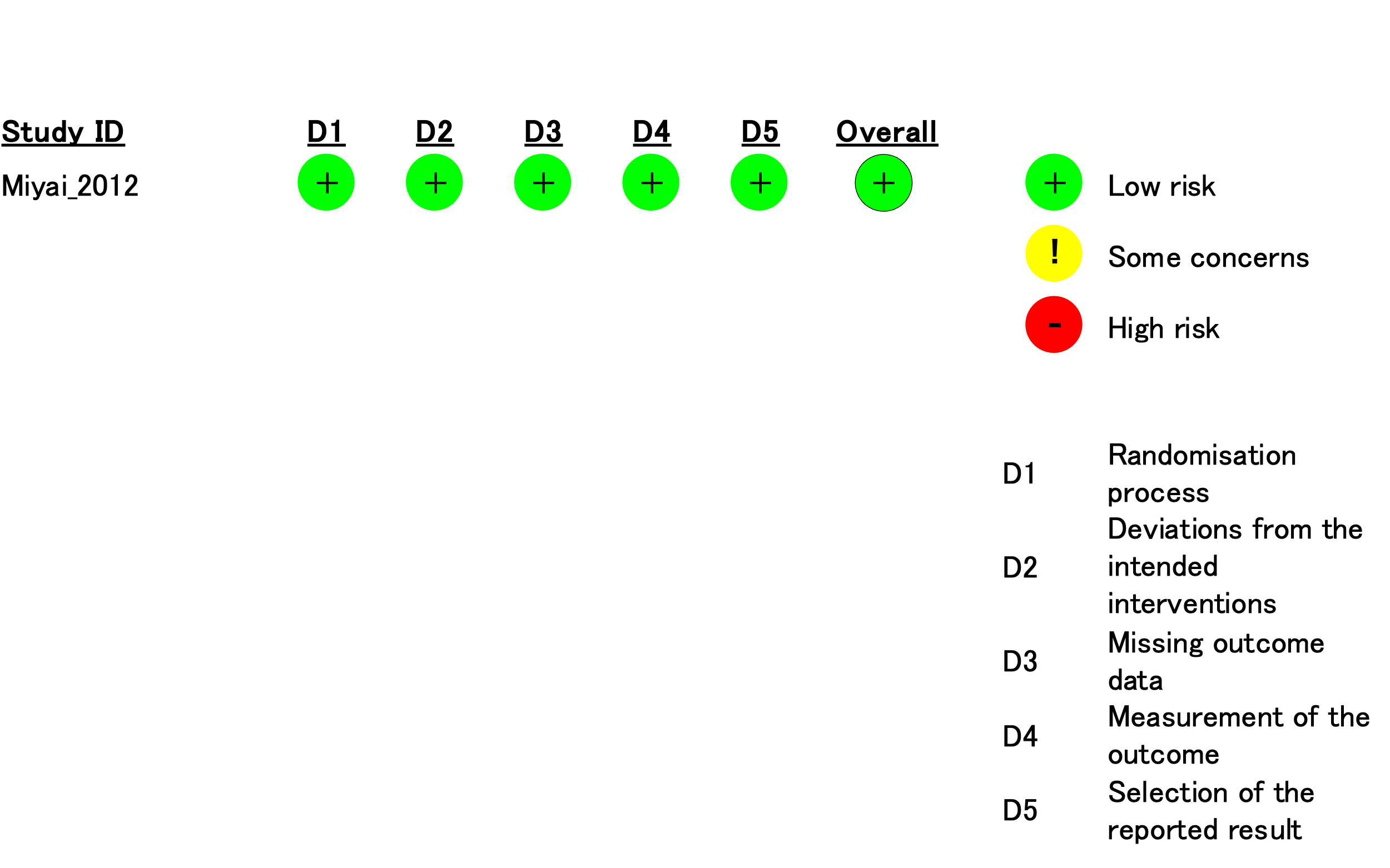


**Supplementary Figure 21.** Risk of bias (RoB) based on fall frequency. “–“ indicates “high RoB,” “!” indicate “some concerns,” and “+” indicates “low RoB.”


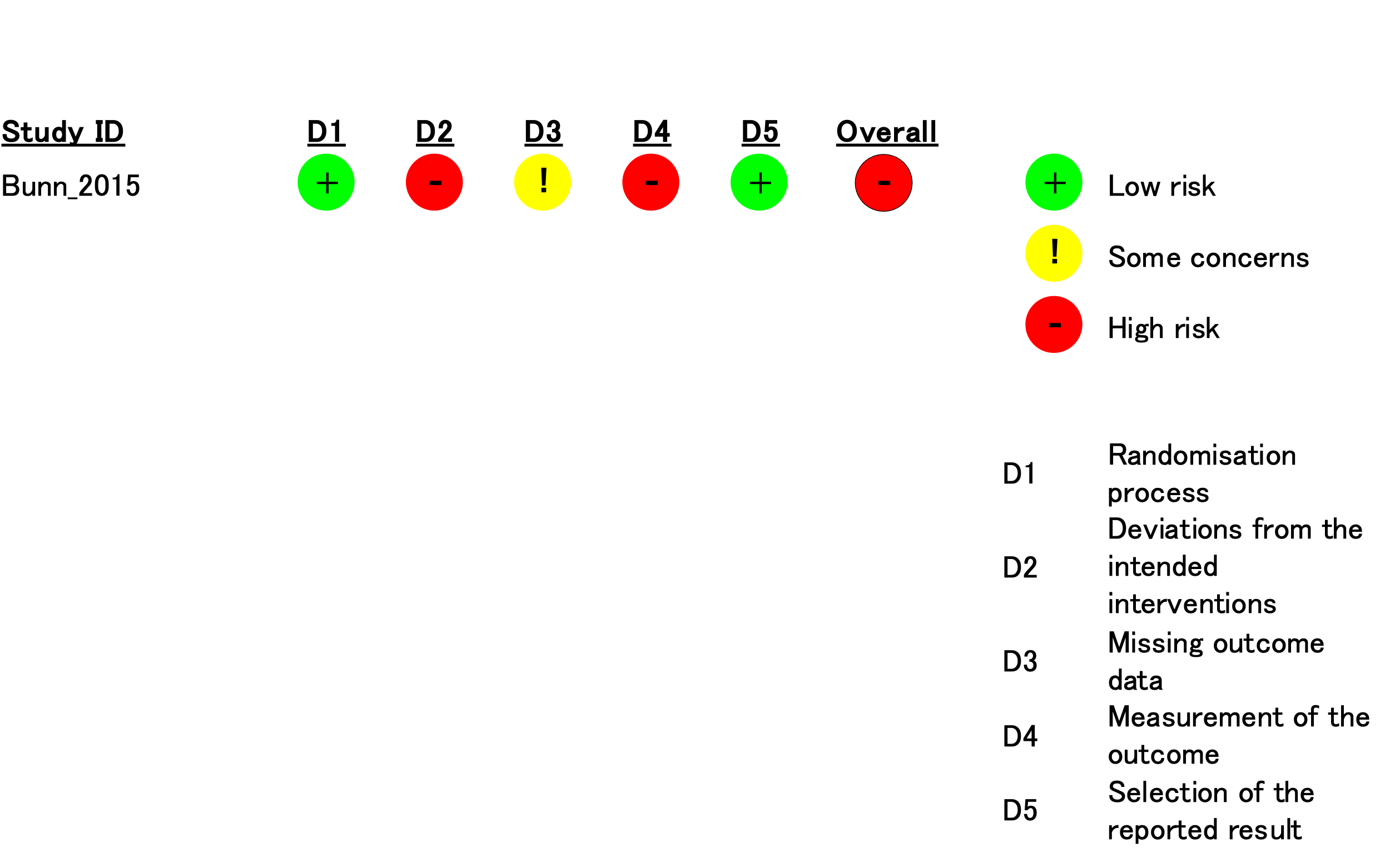


**Supplementary Figure 22.** Risk of bias (RoB) based on the Euro quality of life 5 dimension (EQ-5D). “–“ indicates “high RoB,” “!” indicate “some concerns,” and “+” indicates “low RoB.”


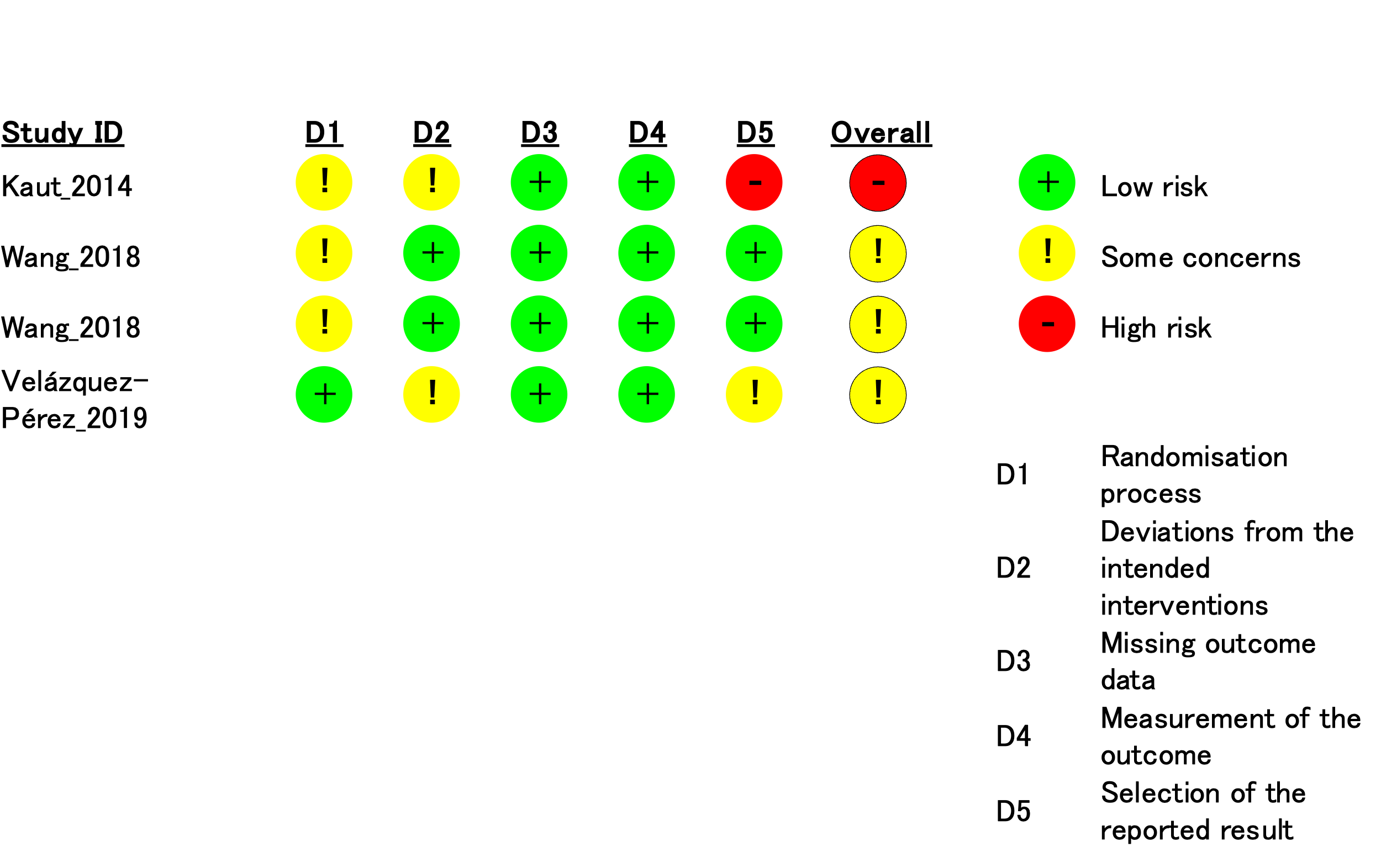


**Supplementary Figure 23.** Risk of bias (RoB) based on the 9 hole peg test (9HPT). “–“ indicates “high RoB,” “!” indicate “some concerns,” and “+” indicates “low RoB.”


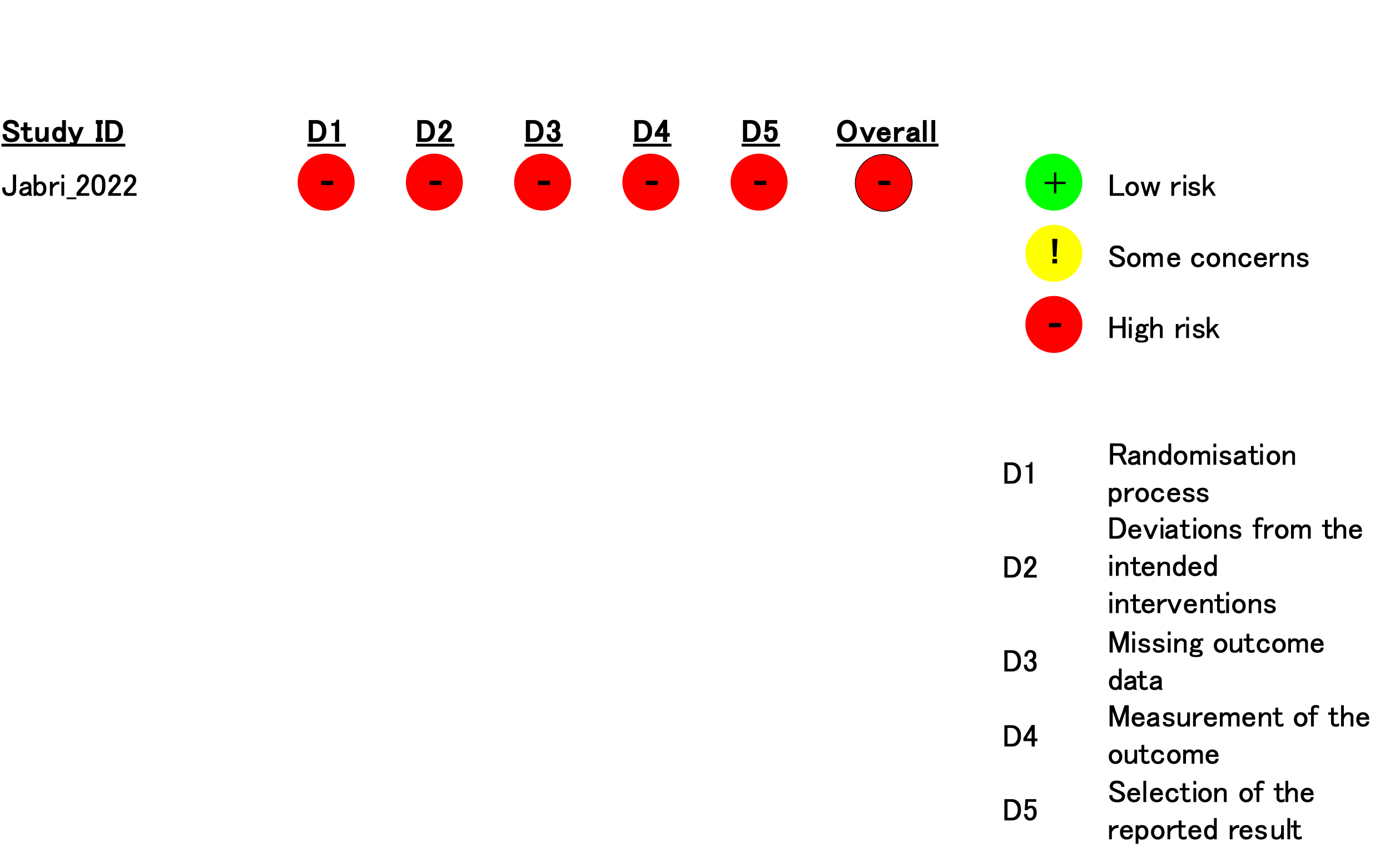


**Supplementary Figure 24.** Risk of bias (RoB) based on the modified Clinical Test of Sensory Interaction in Balance (mCTSIB). “–“ indicates “high RoB,” “!” indicate “some concerns,” and “+” indicates “low RoB.”


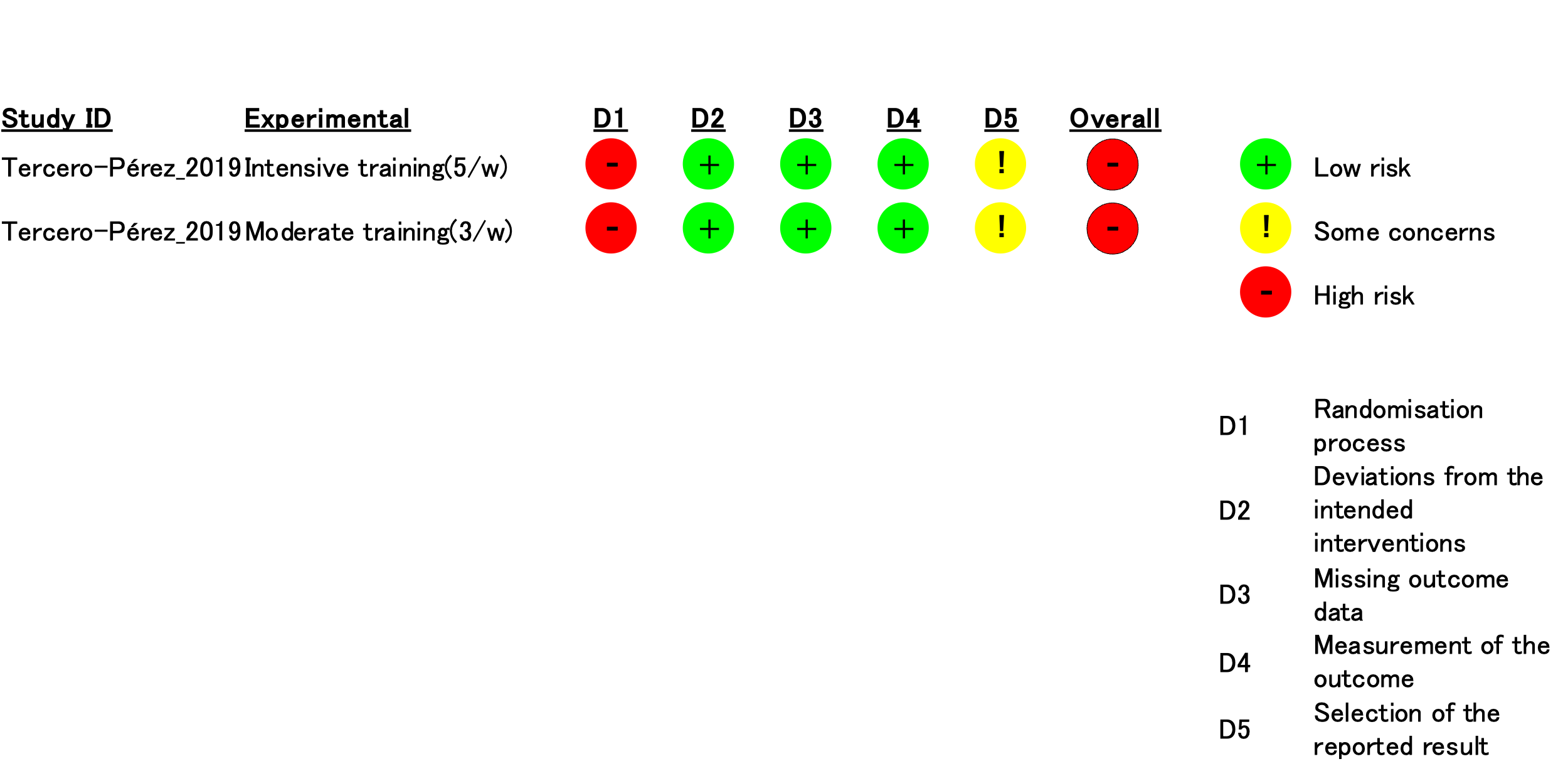


**Supplementary Figure 25.** Risk of bias (RoB) based on the Barthel Index (BI). “–“ indicates “high RoB,” “!” indicate “some concerns,” and “+” indicates “low RoB.”


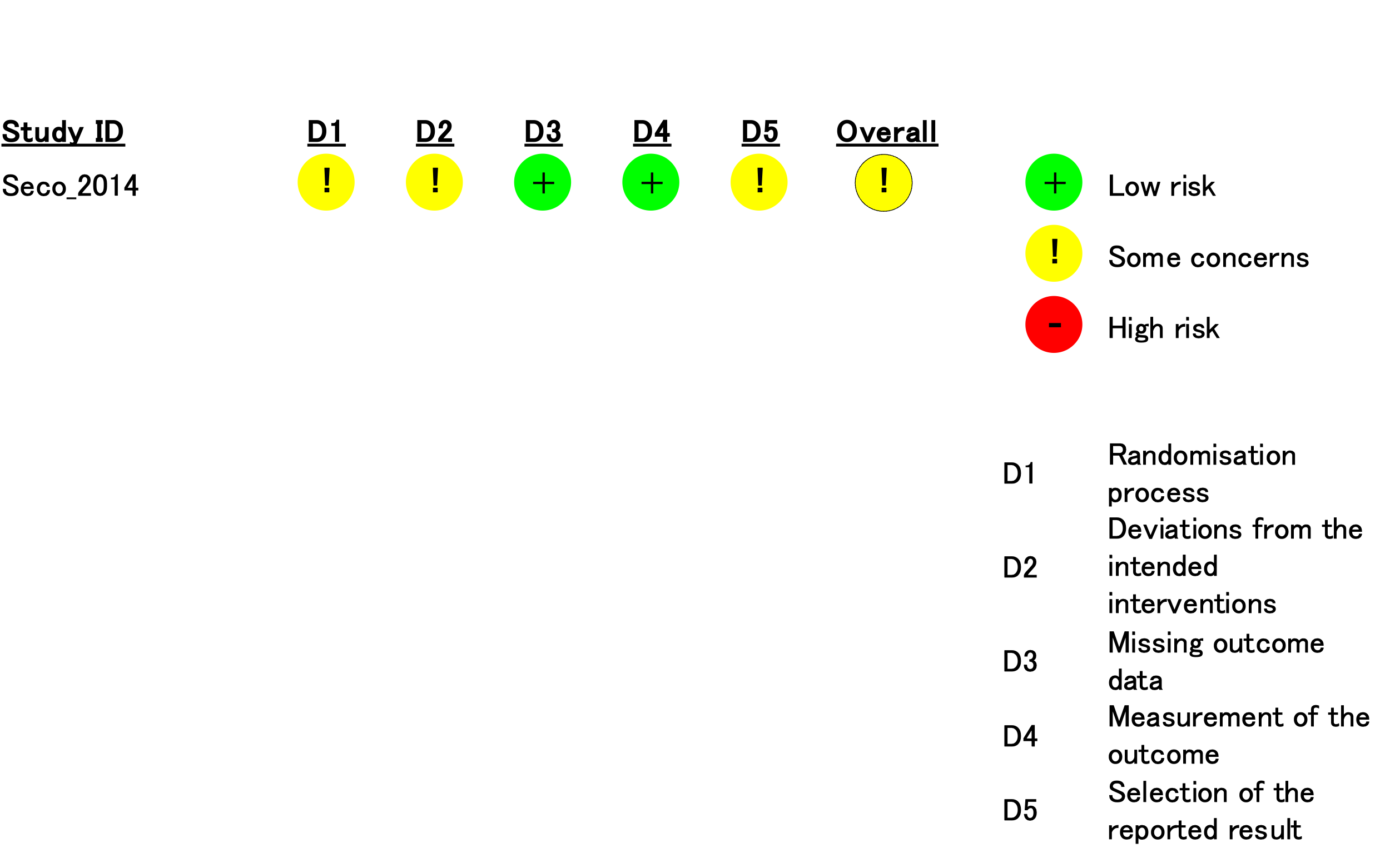


**Supplementary Figure 26.** Risk of bias (RoB) based on the MOS 36-Item Short-Form Health Survey (SF-36). “–“ indicates “high RoB,” “!” indicate “some concerns,” and “+” indicates “low RoB.”

**Appendix1**. Search strategy

PubMed

|  | Search number | Query |
| --- | --- | --- |
| P | 1 | Spinocerebellar Degenerations[mh] OR (Ataxia[mh:noexp] AND (Cerebellum[mh] OR Cerebellar Diseases[mh:noexp])) OR Cerebellar Ataxia[mh] OR ((cerebell*[tiab] OR spinocerebella*[tiab] OR gait[tiab]) AND (atax*[tiab] OR degenerat*[tiab] OR neurodegenerat*[tiab] OR dysmetria*[tiab] OR hemiataxi*[tiab] OR incoordinat*[tiab])) OR "spinocerebellar disease*"[tiab] OR SCA[tiab] OR SCAs[tiab] OR "marinesco syndrome"[tiab:~2] OR "multiple system atroph*"[tiab] OR MSA[tiab] OR "dentatorubral pallidoluysian atroph*"[tiab] OR DRPLA[tiab] OR "wadia syndrome"[tiab:~1] OR "wadia swami"[tiab] |
| I | 2 | "Physical Therapy Technique"[all fields] OR "Physiotherapy"[all fields] OR "Neurological Physiotherapy"[all fields] OR "Neurophysiotherapy"[all fields] OR "physical therapy"[all fields] OR "physiotherapy"[all fields] OR "physical therapist"[all fields] OR "physical therapists"[all fields] OR "physiotherapist"[all fields] OR "physiotherapists"[all fields] OR "physical therapy modalities"[mesh] OR "physical therapy modalities"[all fields] OR "exercise movement techniques"[mesh] OR "exercise movement techniques"[all fields] OR "physical and rehabilitation medicine"[mesh] OR "physical and rehabilitation medicine"[all fields] OR "exercise"[mesh] OR "exercise"[all fields] OR "exercise therapy"[mesh] OR "training"[all fields] OR "trainings"[all fields] OR "neurorehabilitation"[all fields] OR "Robotics"[mesh] or "Robotics"[all fields] or "exoskeleton device"[mesh] or "exoskeleton device"[all fields] OR "aerobic"[all fields] OR "vibration"[all fields] OR “rehabili”[all fields] OR “strength”[all fields] OR "Postural Balance"[mesh] OR "Musculoskeletal Equilibrium"[all fields] OR "Postural Equilibrium"[all fields] OR "balance"[all fields] OR "postural"[all fields] OR "posture"[all fields] OR "coordination"[all fields] OR "coordinations"[all fields] OR "coordinating"[all fields] OR "Resistance Training"[mesh] OR "resistance"[all fields] OR "Strengthening Program"[all fields] OR "Exercise Program"[all fields] OR "Weight Bearing Strengthening Program"[all fields] OR "Weight Bearing Exercise Program"[all fields] OR "Postural Balance"[mesh] OR "Musculoskeletal Equilibrium"[all fields] OR "Postural Equilibrium"[all fields] OR "strength"[all fields] OR "balance"[all fields] OR "postural"[all fields] OR "posture"[all fields] OR "coordination"[all fields] OR "coordinations"[all fields] OR "coordinating"[all fields] OR "treadmill"[all fields] OR "walk"[all fields] OR "walking"[all fields] OR "gait"[all fields] OR "ergometer"[all fields] OR "cycling"[all fields] OR "home"[all fields] OR "out patient"[all fields] |
|  | 3 | #1 AND #2 |
| RCT | 4 | controlled clinical trial[pt] OR randomized[tiab] OR randomised[tiab] OR placebo[tiab] OR clinical trials as topic[mesh:noexp] OR randomly[tiab] OR trial[ti] NOT (animals[mh] NOT humans [mh]) |
|  | 5 | #3 AND #4 |

Cochrane

|  | Search number | Query |
| --- | --- | --- |
| P | 1 | [mh "Spinocerebellar Degenerations"] OR ([mh ^Ataxia] AND ([mh Cerebellum] OR [mh ^"Cerebellar Diseases"])) OR [mh "Cerebellar Ataxia"] OR (((cerebell* OR spinocerebella* OR gait) AND (atax* OR degenerat* OR neurodegenerat* OR dysmetria* OR hemiataxi* OR incoordinat*)) OR (spinocerebellar NEXT disease*) OR SCA OR SCAs OR (marinesco NEAR/3 syndrome) OR ("multiple system" NEXT atroph*) OR MSA OR ("dentatorubral pallidoluysian" NEXT atroph*) OR DRPLA OR (wadia NEAR/2 syndrome) OR "wadia swami"):ti,ab,kw |
| I | 2 | [mh "rehabilitation"] OR (physical OR balance OR gait OR strength OR posture OR aerobic OR coordination OR training OR exercise OR motor OR cognitive OR robotics):ti,ab,kw |
|  | 3 | #1 AND #2 |

CINAHL

|  | Search number | Query |
| --- | --- | --- |
| P | 1 | MH ("Spinocerebellar Degenerations+" OR ("Ataxia" AND ("Cerebellum" OR "Cerebellar Diseases")) OR "Cerebellar Ataxia+")  OR ((TI ("cerebell*" OR "spinocerebella*" OR "gait") OR AB ("cerebell*" OR "spinocerebella*" OR "gait")) AND (TI ("atax*" OR "degenerat*" OR "neurodegenerat*" OR "dysmetria*" OR "hemiataxi*" OR "incoordinat*") OR AB ("atax*" OR "degenerat*" OR "neurodegenerat*" OR "dysmetria*" OR "hemiataxi*" OR "incoordinat*"))) OR TI ("spinocerebellar disease*" OR "SCA" OR "SCAs" OR "multiple system atroph*" OR "MSA" OR "dentatorubral pallidoluysian atroph*" OR "DRPLA" OR "wadia swami" OR ("marinesco" N2 "syndrome") OR ("wadia" N1 "syndrome")) OR AB ("spinocerebellar disease*" OR "SCA" OR "SCAs" OR "multiple system atroph*" OR "MSA" OR "dentatorubral pallidoluysian atroph*" OR "DRPLA" OR "wadia swami" OR ("marinesco" N2 "syndrome") OR ("wadia" N1 "syndrome")) |
| I | 2 |  |
|  | 3 | #1 AND #2 |
| RCT | 4 | (randomized controlled trials OR MH ((random assignment) OR (pretest-posttest design) OR (cluster sample) OR (placebos)) OR TI ((randomised) OR (randomized) OR (trial)) OR AB ((random*) OR (cluster W3 RCT)) OR (MH (sample size) AND AB (assigned OR allocated OR control)) OR PT (randomized controlled trial)) NOT ((MH ((animals+) OR (animal studies)) OR TI (animal model*)) NOT MH human) |
|  | 5 | #3 AND #4 |

PEDro

| Search number | Query |  |
| --- | --- | --- |
| 1 | spinocerebell* AND "clinical trial"   OR  cerebell* AND "clinical trial" |  |
|  |  |  |
|  |  |  |
|  |  |  |
|  |  |  |
